# FACT: a randomised controlled trial to assess the feasibility of QbTest in the assessment process of attention deficit hyperactivity disorder (ADHD) for young people in prison—a feasibility trial protocol

**DOI:** 10.1136/bmjopen-2019-035519

**Published:** 2020-01-20

**Authors:** Charlotte Lennox, Charlotte Lucy Hall, Lesley-Anne Carter, Bryony Beresford, Susan Young, Abdullah Kraam, Nikki Brown, Lloyd Wilkinson-Cunningham, Mindy Reeves, Prathiba Chitsabesan

**Affiliations:** 1 Division of Psychology and Mental Health, The University of Manchester, Manchester, UK; 2 Division of Psychiatry & Applied Psychology, Institute of Mental Health, University of Nottingham, Nottingham, Nottinghamshire, UK; 3 Centre for Biostatistics, Division of Population Health, Health Services Research and Primary Care, The University of Manchester, Manchester, UK; 4 Social Policy Research Unit, University of York, York, North Yorkshire, UK; 5 Department of Clinical and Forensic Psychology, Psychology Services Limited, London, UK; 6 Child and Adolescent Mental Health Service, Rotherham Doncaster and South Humber Mental Health NHS Foundation Trust, Doncaster, UK; 7 Division of Psychiatry & Applied Psychology, Institute of Mental Health, University of Nottingham, Nottingham, Nottinghamshire, UK; 8 Research and Development, Leeds Community Healthcare NHS Trust, Leeds, UK; 9 Medical School, The University of Manchester, Manchester, UK; 10 Child and Adolescent Mental Health Service, Pennine Care NHS Foundation Trust, Ashton-under-Lyne, Lancashire, UK

**Keywords:** child & adolescent psychiatry, forensic psychiatry, clinical trials, qualitative research

## Abstract

**Introduction:**

The prevalence of attention deficit hyperactivity disorder (ADHD) within the Children and Young People Secure Estate (CYPSE) is much higher than seen in the general population. To make a diagnosis of ADHD, clinicians draw on information from multiple sources, including parents and teachers. However, obtaining these is particularly difficult for young people in the secure estate. There is increasing evidence in the community that QbTest is able to assist in the accurate and earlier diagnosis of ADHD. The objective of this study is to assess the feasibility and acceptability of QbTest in the assessment of ADHD within the CYPSE.

**Methods and analysis:**

A single-centre parallel group feasibility randomised controlled trial will be conducted. Sixty young people within the CYPSE identified as displaying possible symptoms of ADHD will be randomised to the intervention arm (n=30; QbTest plus usual care) or control arm (n=30; usual care). Primary analyses will be descriptive and a process evaluation will be conducted to assess the contexts involved in implementing the intervention. Interviews will be conducted to explore acceptability and thematic analysis will be used to analyse the data.

**Ethics and dissemination:**

This study was approved by National Health Service Wales research ethics committee 3 (18/WA/0347) on 15 February 2019. The findings will be published in peer-reviewed journals, presented at relevant conferences and disseminated to the public via summaries cocreated with our patient and public involvement group.

**Trial registration number:**

ISRCTN17402196

**Strengths and limitations of this study**

- This trial is novel as it is one of the first trials within the Children and Young People Secure Estate.
- Understanding the effects of context as barriers or facilitators is critical for interpreting the findings.
- The study will engage meaningfully and substantively with a patient and public involvement group to ensure that the research and intervention are both feasible and acceptable.
- This is a feasibility trial and is not powered to show effectiveness of the intervention.
- This feasibility trial is only being conducted in one site.

## Introduction

In England, children and young people aged 10–17 years old remanded or sentenced into custody are placed in a variety of different secure establishments, known collectively as the Children and Young People Secure Estate (CYPSE). The CYPSE includes: Secure Children’s Homes, Secure Training Centres and Young Offenders Institutions (YOIs). The majority of young people are placed within the YOIs and there are four establishments in England accepting 15–17 years old.

The prevalence rate of attention deficit hyperactivity disorder (ADHD) in the CYPSE is approximately 30%–40%, which is significantly greater than the rate of 5%, seen in the general population.^1 2^ However, a report raised significant concerns about undetected neurodisability, including ADHD within the CYPSE, due to a lack of appropriate screening and assessment processes and training of staff.^3^ Additionally, many young people have comorbid mental health needs contributing to complexity in the assessment process. ADHD is associated with a range of poor outcomes, including a greater risk of developing other mental and physical health needs, educational and occupational problems and offending.^4–13^ The social and economic burden of untreated ADHD on society is significant.^14–19^

The publication of the Healthcare Standards for Children and Young People in Secure Settings^20^ attempted to provide a more standardised approach to the assessment and care provided within the CYPSE. One way was to recommend the use of the Comprehensive Health Assessment Tool (CHAT).^21^ The CHAT was introduced into the CYPSE in 2013 and is completed within all CYPSE sites in England, UK. The CHAT is a semistructured assessment of health needs, delivered by a nurse during the first 10 days of admission. If a nurse from talking with the young person and from observing them believes that they may have a need around difficulty concentrating, restlessness, fidgeting, and so on, then the nurse would recommend in the CHAT care plan that the young person be referred for a full ADHD assessment.

For a formal clinical diagnosis of ADHD, a key aspect is the use of information from informants such as parents, teachers and health professionals.^6 7^ However, access to such information can be difficult to obtain and take a long time to receive, resulting in delays to receiving a confirmed diagnosis. The number of days young people spend in the CYPSE is relatively short.^22^ Therefore, informant information is very often not received before the young person leaves and returns back to the community. There is also evidence from a recent review within the CYPSE that establishments find it difficult to provide interventions to young people who are sentenced to fewer than 6 months, due to delays in assessment and/or diagnosis.^23^ All these factors can lead to delays in young people with ADHD receiving a diagnosis and accessing evidence-based treatment.

The impact of missed opportunities in diagnosis and accessing appropriate treatment can be significant. Young people with untreated ADHD in the CYPSE have been found to have an eightfold increased frequency of aggressive incidents in secure settings.^24 25^ Reducing violence and aggression is a key priority for Her Majesty’s Prison & Probation Service and Ministry of Justice, given the increasing rates within prisons.^26^ Treatment of adult offenders with ADHD using medication has demonstrated a reduction in reoffending of up to 41%.^27^ Therefore, there are also likely social and economic benefits to improving the diagnosis and access to treatment for young people with ADHD in the CYPSE.

QbTest (Qbtech) is a computer task that measures three core aspects of ADHD: attention, impulsivity and motor activity. Performance on the task provides information (via an immediate report) on the three symptom domains of ADHD and a ‘summary score’ based on deviation from a normative data set based on age group and gender. QbTest can be used with individuals with mild learning disabilities as instructions on how to complete the task are visual. Practitioners use the information from the QbTest report in conjunction with the clinical information (patient history, observation and ADHD symptom questionnaire) to inform their decision whether the young person has ADHD or not.

QbTest demonstrates good psychometric properties^28 29^ and has the additional benefit over other computer tasks of measuring activity level, a core symptom of ADHD.^30^ There are several studies that demonstrate the clinical utility of QbTest in aiding diagnosis.^31 32^ A recent randomised controlled trial found clinicians with access to the QbTest report were more likely to reach a diagnostic decision about ADHD. At 6 months, 76% of those with a QbTest report had received a diagnostic decision, compared with 50% without a QbTest report. QbTest reduced appointment length, increased clinicians’ confidence in their diagnostic decisions and doubled the likelihood of excluding ADHD.^33^

The use of the QbTest in the community looks promising.^34^ However, there are a number of unknowns in relation to its use in the CYPSE. Implementing new approaches can require more planning due to the regime; therefore, the potential barriers and facilitators to implementation need to be understood. The young people within the CYPSE are more complex and have greater comorbidity than young people accessing community Child and Adolescent Mental Health Services. The acceptability of the QbTest needs to be assessed to see if the same positive findings can be replicated. Primary outcomes in previous QbTest trials^33^ have been time to diagnosis, but given the comparative short stay in the CYPSE and difficulty obtaining these data once they leave, it is unclear how reliably these data can be collected and/or if other outcomes, such as in aggressive incidents, are more important in this population.

The longer term aim is to undertake a definitive randomised controlled trial of the effectiveness of QbTest in the assessment of ADHD in the CYPSE. An evidence base within the CYPSE needs to be developed in order to change policy and implement the QbTest across the whole system. In line with the Medical Research Council (MRC) guidelines on developing complex interventions and as randomised controlled trials within the CYPSE are scarce, a feasibility study is required.^35^

### Trial objectives

To conduct a pragmatic feasibility randomised controlled trial to assess the feasibility and acceptability of QbTest in the assessment of ADHD within the CYPSE.

### Trial design

A parallel two-group randomised controlled trial with 1:1 individual participant allocation to QbTest plus usual care (n=30; intervention group) or usual care alone (n=30; control group) with a parallel process evaluation. Recruitment started in March 2019 and is due to last 12 months.

## Methods and analysis

### Setting

The study will be conducted in one YOI site within the CYPSE, in England.

### Eligibility criteria

#### Inclusion criteria

- Identified as presenting with ADHD symptoms via the CHAT.
- Aged 15–18 years old.

#### Exclusion criteria

- On remand (uncertain/sudden release).
- Non-English speaking (for pragmatic reasons, but will be included in the main trial testing applicability more widely).
- Previous or current confirmed diagnosis of ADHD.
- Currently receiving ADHD medication.
- Deemed to present risk to either the researcher or the staff.
- Unable to provide informed consent (over 16).
- Parental/legal guardian consent not received (under 16).

Potential participants will be identified by the prison healthcare team. The CHAT will identify potential participants displaying possible symptoms of ADHD, for example, any ADHD symptom codes in CHAT. Sentence status is accessible to healthcare staff via SystmOne (SystmOne is the clinical record system used within the CYPSE) and young people on remand will be excluded. Healthcare records will also be searched for previous or current confirmed diagnosis of ADHD and/or currently receiving ADHD medication and those young people will be excluded. The risk presented by potential participants will be assessed by the healthcare team, based on knowledge of the young person, information contained on SystmOne and discussion with prison staff and will take account of the likely risk in prison and community settings. Potential participants whose risk cannot be managed safely will be excluded.

### Usual care

The CHAT is a semistructured assessment of health needs. It is delivered by a nurse during the first 10 days of admission and includes assessing ADHD needs (not diagnostic) based on clinical history and observation of the young person. Young people identified with potential ADHD on the CHAT are discussed with the psychiatrist at the multidisciplinary team. ADHD questionnaires (self, teacher and parent) are completed and reviewed. If further ADHD assessment is required, this includes clinical assessment (history and observation) and obtaining informant information including developmental history (completed by nurse and psychiatrist). This typically involves a minimum of three appointments with the young person and liaison with teachers/parents.

### Intervention: QbTest plus usual care

Once the young person has been referred to the multidisciplinary team, the young person is booked into the QbTest clinic. The healthcare team will conduct the QbTest and use the information from the QbTest report in conjunction with the clinical information (patient history, observation and ADHD symptom questionnaire) to inform their decision whether the young person has ADHD or not. Young people randomised to the QbTest will receive usual care, as outlined above plus the QbTest. The QbTest takes approximately 20 min to complete and the results will be discussed with the psychiatrist and the young person; this will take approximately 10 min.

### Primary outcomes

- Eligibility rate recorded as the number of eligible young people against the total number of young people identified with ADHD needs.
- Recruitment rate recorded as the number of eligible young people who consent to participate.
- Acceptability of randomisation recorded as the number of young people randomised.
- Acceptability of trial participation recorded as the number of eligible young people who drop out after receiving allocation.
- Attrition rate recorded as the number of young people who consent to participate that remain in the study until the end of follow-up at 6 months.

### Secondary outcomes

- Behaviour is measured using the Strengths and Difficulties Questionnaire (SDQ)^36^ at baseline, 3 and 6 months. The SDQ is a brief behavioural screening questionnaire. It contains 25 items divided between five scales: emotional symptoms; conduct problems; hyperactivity/inattention; peer relationship problems and prosocial behaviour. It takes 5–10 min to complete and is completed with the young person.
- ADHD symptoms are measured using the Brief Barkley ADHD Rating Scale (B-BAARS)^37^ at baseline, 3 and 6 months. The B-BAARS is a newly developed short (six item) screen for ADHD. It takes 5 min to complete and is completed with the young person.
- Health-related quality of life is measured using the Child Health Utility Instrument^38^ at baseline, 3 and 6 months. It takes 5 min to complete and is completed with the young person.
- Objective rating of symptoms and functioning is measured by the Swanson, Nolan and Pelham Questionnaire (SNAP-IV 26)^39^ and Children’s Global Assessment Scale (C-GAS)^40^ at baseline, 3 and 6 months. The SNAP-IV 26 screens for nine symptoms of ADHD hyperactive-impulsive type, nine symptoms of ADHD inattentive type and eight symptoms of oppositional defiant disorder as defined in the Diagnostic and Statistical Manual of Mental Disorders, 4th edition. The SNAP-IV is completed by a teacher within the CYPSE. The C-GAS is a 0–100 scale completed by a clinician which integrates psychological, social and academic functioning.
- Number and duration of (in minutes) consultations/appointments until confirmed ADHD diagnosis as recorded on a pro forma completed by clinicians after each consultation with the young person.
- Number of days until a confirmed diagnosis is reached using file information.
- Contact with services is measured using the modified Client Service Receipt Inventory^41^ at baseline, 3 and 6 months. It takes 5–10 min to complete and is completed with the young person.
- Number of reordered behavioural incidents and adjudications are collecting using prison file information at baseline, 3 and 6 months.

### Sample size and recruitment

The required sample is 60 young people, 30 per study arm. This is large enough to test the feasibility of the research procedures and to establish a mean and SD on each outcome measure.^42^ Five young people are to be randomised into the study each month. Recruitment rates and the final target will be used to inform the decision to proceed to a definitive trial.

Attrition will be minimised by having robust trial procedures to prevent data loss. Procedures have been developed with our patient and public involvement (PPI) groups and tested for maintaining contact with participants. Recognising that this population can be difficult to follow-up, follow-up data collection points will occur within broad time windows. Having very broad time windows allows for as many follow-ups to be completed within the CYPSE, but also ensuring that no young people are excluded from the study if they miss a follow-up.

### Randomisation and blinding

Randomisation will be achieved by means of concealed random allocation conducted using an online pseudorandom list hosted by Sealedenvelope.com with random permuted blocks of varying sizes. Randomisation will be undertaken by the researcher, who will inform healthcare staff of trial arm allocation.

Given the nature of the intervention, it is not possible to blind participants or those involved in delivering the intervention. It is also not possible to blind the field researcher due to the researcher and staff delivering the intervention working within the confines of a secure environment. It is also not possible to use a second researcher to collect follow-up data (who does not know trial arm allocation) as this is logistically very challenging and ultimately conflicts with the idea of building a strong researcher–participant relationship to enhance follow-up rates. Therefore, to minimise any potential bias, protocolising the collection of outcome measures which will be overseen by the onsite principal investigator and trial manager (CL). None of the outcome measures completed by the young person with the researcher present are particularly susceptible to bias as they are standardised, self-complete questionnaires. The researcher may need to support the young person in completing questionnaires due to literacy levels and protocolising collection ensures that support is consistent and unbiased. The study statistician (L-AC) will be blind to arm allocation.

#### Data collection

See table 1 for a summary of the study schedule of data collection.

**Table 1:** Summary of study schedule

Timepoint		Screening	Baseline	Allocation	Follow-up	
		t0	t1		+3 mth t2	+6 mth t3
Enrolment:						
Eligibility screen		X				
Informed consent		X				
*Parental consent (* *<* *16)*		X				
Allocation				X		
Interventions:						
*Intervention group:*	*QbTest*	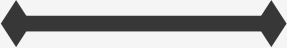				
	Usual care	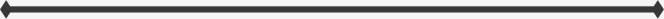				
*Control group:*	*Usual care*	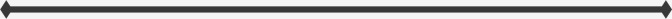				
Assessments:						
*Demographics*			X		X	
*SDQ*			X		X	
*B-BAARS*			X		X	
*CHU-9D*			X		X	
*SNAP-IV*			X		X	
*C-GAS*			X		X	
*ADHD diagnosis* *and* *clinical confidence*					X	
*Health records*			X		X	
*Prison records*			X		X	
*Modified CSRI*			X		X	
*Contact sheet*			X		X	
Safety monitoring:						
Adverse event reporting		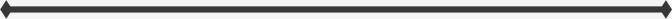				

ADHD, attention deficit hyperactivity disorder; B-BAARS, Brief Barkley ADHD Rating Scale; C-GAS, Children's Global Assessment Scale; CHU-9D, Child Health Utility Instrument; CSRI, Client Service Receipt Inventory; SDQ, Strengths and Difficulties Questionnaire; SNAP-IV, Swanson, Nolan and Pelham Questionnaire.

#### Baseline data collection (t1)

The researcher will deliver the baseline data collection interview using a narrative conversational format. Demographics and questions from other measures are incorporated into a specially constructed flexible paper case report form which avoids duplication of subject matter in order to reduce disengagement or irritability.

#### Follow-up (t2–t3)

If the young person is to be released during the study, the researcher will meet with them usually within the week prior to release. During this meeting, the researcher will confirm the contact information provided at baseline and make any amendments to the information (eg, change of phone number). Once discharged, the researcher will contact the participant again in the community, to maintain engagement, confirm contact details and arrange the follow-up in the community.

The 3-month follow-up can take place between 61 and 151 days post randomisation, although the researcher will endeavour to complete data collection as close to the 3-month (90-day) point. The 6-month follow-up can take place between 152 and 244 days postrandomisation, although the researcher will endeavour to complete data collection close to the 6-month point (182 days).

The follow-up may take place in prison or in the community, depending on if the young person has been discharged. In the community, the researcher will arrange to meet the young person at a convenient location. Where possible, interviews will be conducted in the premises of services that the young person is engaged with in order to minimise risk to the researcher. Where this is not possible, the researcher will arrange to conduct the interviews in a suitable location in the community and adhere to the Local Lone Working Policy.

### Process evaluation

QbTest in itself may not be defined as a complex intervention requiring a process evaluation to understand the mechanisms of impact, for example, how QbTest produces change; however, delivering an intervention within a criminal justice setting is complex. Understanding the effects of the context as barriers or facilitators is critical for interpreting the findings and generalising beyond it to a full trial. The process evaluation will follow the MRC guidelines^43^ and will be conducted in parallel to the trial and will assess:

- the acceptability of randomisation (interviews);
- the acceptability of outcome measures (completeness of each measure and interviews);
- the numbers of young people agreeing to sit the QbTest and the number who complete QbTest;
- feedback from young people receiving the QbTest and health professionals using a questionnaire;
- the acceptability of the QbTest via interviews with young people; and
- health professionals and other CYPSE staff to obtain perspectives on facilitators and barriers to using the QbTest.

### Data collection

QbTest completion rates: a record will be taken of the numbers of participants who agree to sit and also complete the QbTest.

QbTest opinion questionnaire: all young people who receive QbTest will be asked to complete the QbTest opinion questionnaire at 3 months. The questionnaire contains 11 questions, for example, ‘the QbTest results were difficult to understand’ and the young person is asked to rate each item on a 5-point scale. At month 15, clinicians involved in the QbTest will be asked to complete the clinician version, which contains 13 questions rated on a 5-point scale and two free-text response questions.^29^

Interviews with young people: randomisation: a subsample of young people in both trial arms will be interviewed using a semistructured interview schedule to further explore acceptability of the randomisation process. Young person’s age and time of randomisation (beginning or end of study) will be used to purposively select the sample. Outcomes: a subsample of young people in both trial arms will be interviewed using a semistructured interview schedule to further explore acceptability of completion of the outcome measures. Young person’s age and time of randomisation (beginning or end of study) will be used to purposively select the sample. QbTest: a subsample of young people who receive QbTest will be interviewed using a semistructured interview schedule to further explore acceptability of the QbTest and its administration as well as study processes and procedures. Scores/responses in the QbTest opinion questionnaire, young person’s age and whether or not the QbTest was completed will be used to purposively select the sample. Up to 20 young people will be interviewed.

Interviews with healthcare professionals and CYPSE staff: healthcare professionals and CYPSE staff will be invited to participate in qualitative interviews to further explore acceptability and feasibility of administering and implementing the QbTest within usual assessment practices and procedures, including the barriers to its use and reasons for non-completion. Scores/responses in the QbTest Opinion Questionnaire will be used to purposively select healthcare professionals. Up to 10 professionals will be interviewed.

### Data analysis and management

Data analysis will be mainly descriptive.^44^ All measures will be summarised by group across follow-up time with mean and SD for normally distributed data, median for skewed variables and frequency (percentage) for categorical data. All statistical analysis will be conducted using Stata V.15. No interim analysis is planned.

Interviews will be conducted and recorded by the researcher using an encrypted Dictaphone. All qualitative data will be analysed using thematic analysis^45^ with the aid of NVivo. Data will be coded inductively into themes, creating a detailed coding scheme, allowing for the investigation of emergent patterns between individual codes and boarder emergent themes.

Data will be double entered and stored on an encrypted and password-protected database at the University of Manchester. Double-entered data will be compared for discrepancies and discrepant data will be verified using the original paper data sheets (between LW-C and CL).

### Monitoring

#### Adverse events

Serious adverse events (SAEs) will be recorded by the research team and reported to the chief investigator and Trial Steering Committee (TSC). Any SAEs deemed to have a causal relationship to trial participation will be reported to the sponsor within 24 hours of the chief investigator being informed.

#### Trial management and oversight

The TSC will oversee the conduct and safety of the trial and includes the role of the data monitoring. The committee includes an independent chair, independent members, a patient and public representative and the chief investigator. Representatives from both the sponsor and funding organisations will be invited to study-related elements of the TSC meetings as observers. The TSC will meet quarterly.

#### Audit

The trial coordinator or a nominated designee of the sponsor shall carry out monitoring of trial data as an ongoing activity. Trial data and evidence of monitoring and audits will be made available for inspection by the research ethics committee as required.

## Ethics and dissemination

Health research authority approvals have been granted. Only the research team will have access to the study data, which will be stored in secure locked files or password-protected databases. Data will be available for inspection by the ethics committee on request. Changes to the protocol will be communicated to the ethics committee and trial registries by the trial manager (CL).

### Consent process

Obtaining informed consent or assent will be in accordance with ethical guidance and good clinical practice. The researcher will be trained to assess for capacity to consent in young people. This will include assessing if the young person has the ability to understand the information relevant to the decision, retain the information, use or weigh the information as part of the process for decision-making and communicate the decision to the researcher.

The researcher will explain what participation in the study involves and how much time will be involved. They will also explain that participation is voluntary, that they can withdraw at any time and at any point and that their decision to participate, or not, will have no adverse effect on the care that they receive or their other legal rights. The researcher will also discuss the arrangements in place to ensure confidentiality (and limits of this) and data protection. Throughout this process, the potential participant will be given an opportunity to ask questions. Potential participants will be made aware of circumstances in which confidentiality would be broken (if they or someone else identified may be at potential risk of harm; for example, self-harm, breaches of security and violence, including acts of terrorism/radicalisation).

Having had the opportunity to discuss their involvement in the study and ask questions about it, potential participants will be asked if they would like time (minimum 24 hours) to consider taking part or if they would like to sign the consent form (online supplementary file 1—consent form) if they are willing to take part.

10.1136/bmjopen-2019-035519.supp1 Supplementary data

### Patient and Public Involvement

PPI significantly informs and impacts on this project. NB (coapplicant and coauthor) is the project PPI representative and is directly involved in the project providing her lived experience expertise throughout the project ensuring that the perspective of young people with ADHD and their parents are considered throughout. The project has PPI representatives from the community and also within the CYPSE and both groups have helped develop the information leaflets for the study and also helped develop and refine research procedures, for example, how best to explain what participation in the study involves and how best to achieve community follow-ups. The CYPSE PPI group meet with the researcher (LW-C) on a regular basis to feed into the project. The PPI groups will be involved in the analysis, reporting and dissemination of the research.

### Dissemination

The findings from the trial will be used to inform the design, feasibility and acceptability of a future definitive trial. The findings will be published in peer-reviewed journals, presented at relevant conferences and disseminated to the public via summaries cocreated with our PPI group.

## Discussion

Secure settings are complex environments in which to conduct trials^46^ and there is a dearth of trials within the CYPSE. In the context of this challenging environment, a decision will be taken as to whether feasibility and acceptability are adequate for a full multicentre randomised controlled trial.

Current service provision for ADHD in the CYPSE is ad hoc and uncoordinated. There is growing evidence within community settings^30 32–34^ that QbTest has the potential to significantly improve the current assessment pathway having a direct impact on young people’s health, service delivery and criminal justice outcome.

## Supplementary Material

Reviewer comments

Author's manuscript
