# Supplementary material for: FACT: a randomised controlled trial to assess the feasibility of QbTest in the assessment process of attention deficit hyperactivity disorder (ADHD) for young people in prison—a feasibility trial protocol
Source: BMJ Open. 2020 Jan 20;10(1):e035519. doi: 10.1136/bmjopen-2019-035519 (PMC7044874; doi:10.1136/bmjopen-2019-035519)
Supplement: Supplementary data [file bmjopen-2019-035519supp001.pdf]

Pennine Care NHS Foundation Trust

Leeds Community Healthcare NHS Trust

FACT: Feasibility trial to assess ADHD in the Criminal Justice System via QBTest

PARTICIPANT CONSENT FORM

IRAS project ID: 238947

Please **initial**

1. I confirm that I have read the attached information sheet (**Version X, Date: dd/mm/yy**). ☐

2 I have had time to think about the information, ask questions and had these answered. ☐

3. I understand that taking part in the study is up to me and that I am free to stop taking part at any time without giving a reason. This will not affect my care or cause problems for me. ☐

4. I understand that the researcher has to tell prison staff should I talk about: ☐
   a) Serious behaviour that is against prison rules;
   b) Harm to myself or others;
   c) Plans to commit new crimes or past crimes that the police do not know about;
   d) Information that raises concerns about terrorist, radicalisations, or security issues.

5. I understand that I will be in the QbTest group or usual care group. My group will be chosen at random, and I won't be able to choose. ☐

6. I agree to the research team looking at my criminal justice and health records, but only for the purpose of this research project. ☐

7. I agree to interviews being audio recorded. ☐

8. I agree that direct quotes can be used in any study write-up, in anonymous form. ☐

9. I agree that any data collected may be published in anonymous form in academic books, reports or journals ☐

10. I agree that the researcher team can have my contact details so that they can contact me for the 3 and 6 month follow up interviews. ☐

11. I agree to take part in this study ☐

Sign below for participant completed consent form

Name of participant	Date	Signature
Name of researcher	Date	Signature

(Version 2, Date: 25.10.18) 1

Pennine Care NHS Foundation Trust

Leeds Community Healthcare NHS Trust

*1 copy for participant: 1 copy for researcher: 1 copy for the participant record

Data Protection - The personal information we collect and use to conduct this research will be processed in accordance with data protection law as explained in the Participant Information Sheet.

Lennox C, *et al. BMJ Open* 2020; 10:e035519. doi: 10.1136/bmjopen-2019-035519
