# Supplementary material for: FACT: a randomised controlled trial to assess the feasibility of QbTest in the assessment process of attention deficit hyperactivity disorder (ADHD) for young people in prison—a feasibility trial protocol
Source: BMJ Open. 2020 Jan 20;10(1):e035519. doi: 10.1136/bmjopen-2019-035519 (PMC7044874; doi:10.1136/bmjopen-2019-035519)
Supplement: Author's manuscript [file bmjopen-2019-035519.draft_revisions.pdf]

BMJ Open is committed to open peer review. As part of this commitment we make the peer review history of every article we publish publicly available.

When an article is published we post the peer reviewers' comments and the authors' responses online. We also post the versions of the paper that were used during peer review. These are the versions that the peer review comments apply to.

The versions of the paper that follow are the versions that were submitted during the peer review process. They are not the versions of record or the final published versions. They should not be cited or distributed as the published version of this manuscript.

BMJ Open is an open access journal and the full, final, typeset and author-corrected version of record of the manuscript is available on our site with no access controls, subscription charges or pay-per-view fees (http://bmjopen.bmj.com).

If you have any questions on BMJ Open's open peer review process please email editorial.bmjopen@bmj.com

BMJ Open

FACT: A randomised controlled trial to assess the feasibility of QbTest in the assessment process of ADHD for young people in prison: A feasibility trial protocol.

Journal:	*BMJ Open*
Manuscript ID	bmjopen-2019-035519
Article Type:	Protocol
Date Submitted by the Author:	05-Nov-2019
Complete List of Authors:	Lennox, Charlotte; The University of Manchester, Division of Psychology and Mental Health<br>Hall, Charlotte; University of Nottingham, Division of Psychiatry & Applied Psychology, Institute of Mental Health,<br>Carter, Lesley-Anne; The University of Manchester, Centre for Biostatistics, Division of Population Health, Health Services Research and Primary Care<br>Beresford, Bryony; University of York, Social Policy Research Unit<br>Young, Susan; Psychology Services Limited<br>Kraam, Abdullah ; Rotherham NHS Foundation Trust<br>Brown, Nikki; University of Nottingham<br>Wilkinson-Cunningham, Lloyd; Leeds Community Healthcare NHS Trust<br>Reeves, Mindy; The University of Manchester<br>Chitsabesan, Prathiba; Pennine Care NHS Foundation Trust
Keywords:	Child & adolescent psychiatry < PSYCHIATRY, Forensic psychiatry < PSYCHIATRY, Clinical trials < THERAPEUTICS, QUALITATIVE RESEARCH

SCHOLARONE™
Manuscripts

FACT: A randomised controlled trial to assess the feasibility of QbTest in the assessment process of ADHD for young people in prison: A feasibility trial protocol.

C Lennox[1]; C L Hall; L.A Carter; B Beresford; S Young; A Kraam; N Brown; L Wilkinson-Cunningham; M Reeves; P Chitsabesan

[1]Dr Charlotte Lennox (Corresponding author); Division of Psychology and Mental Health, School of Health Sciences, Room 2.315 Jean McFarlane Building, The University of Manchester, Oxford Road, Manchester, M13 9PL charlotte.lennox@manchester.ac.uk

Dr Charlotte L Hall, Division of Psychiatry & Applied Psychology, Institute of Mental Health, University of Nottingham Innovation Park, Triumph Road, Nottingham, NG7 2TU, charlotte.hall@nottingham.ac.uk

Dr Lesley-Anne Carter, Centre for Biostatistics, Division of Population Health, Health Services Research and Primary Care, University of Manchester, 1.304 Jean McFarlane Building, University of Manchester, Oxford Road, Manchester, M13 9PL lesley-anne.carter@manchester.ac.uk

Professor Bryony Beresford, Social Policy Research Unit, University of York, Heslington, York, YO10 5DD, bryony.beresford@york.ac.uk

Professor Susan Young, Psychology Services Limited, London, UK suzyyoung@aol.com

Dr Abdullah Kraam, Leeds Community Healthcare, HMPYOI Wetherby, York Rd, Wetherby LS22 5ED, a.kraam@nhs.net

Nikki Brown, Patient and Public Involvement (PPI) Representative, nikki.l.brown@sky.com

For peer review only - http://bmjopen.bmj.com/site/about/guidelines.xhtml

Lloyd Wilkinson-Cunningham, Leeds Community Healthcare, HMPYOI Wetherby, York Rd, Wetherby LS22 5ED, lloyd.wilkinson-cunningham@nhs.net

Dr Mindy Reeves, Specialty Trainee Doctor in Forensic Child and Adolescent Psychiatry, University of Manchester, mindy.reeves@doctors.org.uk

Dr Prathiba Chitsabesan, Pennine Care NHS Foundation Trust, 225 Old St, Ashton-under-Lyne OL6 7SR, pchitsabesan@nhs.net

For peer review only

ABSTRACT

Introduction: The prevalence of ADHD within the Children and Young People Secure Estate (CYPSE) is much higher than seen in the general population. To make a diagnosis of ADHD clinicians draw on information from multiple sources, including parents and teachers. However, obtaining these is particularly difficult for young people in the secure estate. There is increasing evidence in the community that QbTest is able to assist in the accurate and earlier diagnosis of ADHD. The objective of this study is assess the feasibility and acceptability of QbTest in the assessment of ADHD within the CYPSE.

Methods and Analysis: Single centre parallel group feasibility randomised controlled trial. Sixty young people within the CYPSE identified as displaying possible symptoms of ADHD will be randomised to the intervention arm (n = 30; QbTest plus usual care) or control arm (n = 30; usual care). Primary analyses will be descriptive and a process evaluation will be conducted to assess the contexts involved in implementing the intervention. Interviews will be conducted to explore acceptability and thematic analysis will be used to analyse the data.

Ethics and Dissemination: This study was approved by NHS Wales REC 3 (18/WA/0347) on 15th February 2019. The findings will be published in peer-reviewed journals, presented at relevant conferences and disseminated to the public via summaries co-created with our Patient and Public Involvement group.

Trial registration: ISRCTN registry ISRCTN17402196

For peer review only

Article Summary

Strengths and limitations of this study

- This study will be one of the first in trials within the CYPSE.
- The study will engage meaningfully and substantively with a patient and public involvement group to ensure the research and intervention are both feasible and acceptable.
- This is a feasibility trial and is not powered to show effectiveness of the intervention.

For peer review only - http://bmjopen.bmj.com/site/about/guidelines.xhtml

Keywords: Child & adolescent psychiatry, Forensic psychiatry, Clinical trials, Qualitative research

Word Count: 3990

INTRODUCTION

In England children and young people aged 10-17 years old, remanded or sentenced into custody are placed in a variety of different secure establishments, known collectively as the Children and Young People Secure Estate (CYPSE). The CYPSE includes: Secure Children's Homes (SCHs), Secure Training Centres (STCs) and Young Offenders Institutions (YOIs). The majority of young people are placed within the YOIs and there are four establishments in England accepting 15–17 year olds.

The prevalence rate of attention deficit hyperactivity disorder (ADHD) in the CYPSE is approximately 30-40%, which is significantly greater than the rate of 5%, seen in the general population [1, 2]. However, a report raised significant concerns about undetected neurodisability, including ADHD within the CYPSE, due to a lack of appropriate screening and assessment processes and training of staff [3]. Additionally, many young people have co-morbid mental health needs contributing to complexity in the assessment process. ADHD is associated with a range of poor outcomes, including a greater risk of developing other mental and physical health needs, educational and occupational problems and offending [4-13]. The social and economic burden of untreated ADHD on society is significant [14-19].

The publication of the Healthcare Standards for Children and Young People in Secure Settings [20] attempted to provide a more standardised approach to the assessment and care provided within the CYPSE. One way was to recommend the use of the Comprehensive Health Assessment Tool (CHAT) [21]. The CHAT was introduced into the CYPSE in 2013 and is completed within all CYPSE sites in England, UK. The CHAT is a semi-structured assessment of health needs, delivered by a nurse during the first 10 days of admission. If a nurse from talking with the young person and from observing them believes that they may have a need around difficulty concentrating, restlessness, fidgeting etc. then

For peer review only - http://bmjopen.bmj.com/site/about/guidelines.xhtml

the nurse would recommend in the CHAT care plan that the young person be referred for a full ADHD assessment.

For a formal clinical diagnosis of ADHD, a key aspect is the use of information from informants such as parents, teachers and health professionals [6-7]. However, access to such information can be difficult to obtain and take a long time to receive, resulting in delays to receiving a confirmed diagnosis. The number of days young people spend in the CYPSE is relatively short [22]. Therefore, informant information is very often not received before the young person leaves and returns back to the community. There is also evidence from a recent review within the CYPSE that establishments find it difficult to provide interventions to young people who are sentenced to fewer than six months, due to delays in assessment and/or diagnosis [23]. All these factors can lead to delays in young people with ADHD receiving a diagnosis and accessing evidence based treatment.

The impact of missed opportunities in diagnosis and accessing appropriate treatment can be significant. Young people with untreated ADHD in the CYPSE have been found to have an eight fold increased frequency of aggressive incidents in secure settings [24, 25]. Reducing violence and aggression is a key priority for Her Majesty's Prison & Probation Service (HMPPS) and Ministry of Justice, given the increasing rates within prisons [26]. Treatment of adult offenders with ADHD using medication has demonstrated a reduction in re-offending of up to 41% [27]. Therefore, there are also likely social and economic benefits to improving the diagnosis and access to treatment for young people with ADHD in the CYPSE.

QbTest (Qbtech Ltd) is a computer task that measures three core aspects of ADHD: attention, impulsivity and motor activity. Performance on the task provides information (via an immediate report) on the three symptom domains of ADHD and a 'summary score' based on deviation from a normative data set based on age group and gender. QbTest can be used with individuals with mild learning disabilities as instructions on how to complete the task are visual. Practitioners use the information from the QbTest report in conjunction with

the clinical information (patient history, observation and ADHD symptom questionnaire) to inform their decision whether the young person has ADHD or not.

QbTest demonstrates good psychometric properties [28, 29] and has the additional benefit over other computer tasks of measuring activity level, a core symptom of ADHD [30]. There are several studies that demonstrate the clinical utility of QbTest in aiding diagnosis [31, 32]. A recent randomised controlled trial found clinicians with access to the QbTest report were more likely to reach a diagnostic decision about ADHD. At 6-months, 76% of those with a QbTest report had received a diagnostic decision, compared with 50% without a QbTest report. QbTest reduced appointment length, increased clinicians' confidence in their diagnostic decisions and doubled the likelihood of excluding ADHD [33].

The use of the QbTest in the community looks promising [34]. However, there are a number of unknowns in relation to its use in the CYPSE. Implementing new approaches can require more planning due to the regime; therefore the potential barriers and facilitators to implementation need to be understood. The young people within the CYPSE are more complex and have greater co-morbidity than young people accessing community Child and Adolescent Mental Health Services. The acceptability of the QbTest needs to be assessed to see if the same positive findings can be replicated. Primary outcomes in previous QbTest trials [33] have been time to diagnosis, but given the comparative short stay in the CYPSE and difficulty obtaining this data once they leave, it is unclear how reliably this data can be collected and/or if other outcomes, such as in aggressive incidents are more important in this population.

The longer term aim is to undertake a definitive randomised controlled trial of the effectiveness of QbTest in the assessment of ADHD in the CYPSE. An evidence base within the CYPSE needs to be developed in order to change policy and implement the QbTest across the whole system. In line with the Medical Research Council guidelines on

For peer review only - http://bmjopen.bmj.com/site/about/guidelines.xhtml

developing complex interventions and as randomised controlled trials within the CYPSE are scarce, a feasibility study is required [35].

Trial Objectives

To conduct a pragmatic feasibility randomised controlled trial to assess the feasibility and acceptability of QbTest in the assessment of ADHD within the CYPSE.

Trial Design

A parallel two group randomised controlled trial with 1:1 individual participant allocation to QbTest plus usual care (n = 30; intervention group) or usual care alone (n = 30; control group) with a parallel process evaluation. Recruitment started in March 2019 and is due to last 12 months.

METHODS and ANALYSIS

Setting

The study will be conducted in one YOI site within the CYPSE, in England.

Eligibility Criteria

Inclusion criteria:

- Identified as presenting with ADHD symptoms via the CHAT; and
- Aged 15-18 years old.

Exclusion criteria:

- On remand (uncertain/sudden release);
- Non-English speaking (for pragmatic reasons, but will be included in the main trial testing applicability more widely);
- Previous or current confirmed diagnosis of ADHD;
- Currently receiving ADHD medication;
- Deemed to present risk to either the researcher or the staff;

For peer review only - http://bmjopen.bmj.com/site/about/guidelines.xhtml

- Unable to provide informed consent (over 16); and
- Parental/legal guardian consent not received (under 16)

Potential participants will be identified by the prison healthcare team. The CHAT will identify potential participants displaying possible symptoms of ADHD e.g. any ADHD symptom codes in CHAT. Sentence status is accessible to healthcare staff via SystmOne[1] and young people on remand will be excluded. Healthcare records will also be searched for previous or current confirmed diagnosis of ADHD and/or currently receiving ADHD medication and those young people will be excluded. The risk presented by potential participants will be assessed by the healthcare team, based on knowledge of the young person, information contained on SystmOne and discussion with prison staff and will take account of the likely risk in prison and community settings. Potential participants whose risk cannot be managed safely will be excluded.

Usual Care

The CHAT is a semi-structured assessment of health needs. It is delivered by a nurse during the first 10 days of admission and includes assessing ADHD needs (not diagnostic) based on clinical history and observation of the young person. Young people identified with potential ADHD on the CHAT are discussed with the Psychiatrist at the Multi-Disciplinary Team. ADHD questionnaires (self, teacher and parent) are completed and reviewed. If further ADHD assessment is required, this includes clinical assessment (history and observation) and obtaining informant information including developmental history (completed by nurse and psychiatrist). This typically involves a minimum of three appointments with the young person and liaison with teachers/parents.

Intervention – QbTest plus usual care

Once the young person has been referred to the Multi-Disciplinary Team the young person is booked into the QbTest clinic. The healthcare team will conduct the QbTest and use the information from the QbTest report in conjunction with the clinical information (patient

[1] SystmOne is the clinical record system used within the CYPSE

For peer review only - http://bmjopen.bmj.com/site/about/guidelines.xhtml

history, observation and ADHD symptom questionnaire) to inform their decision whether the young person has ADHD or not. Young people randomised to the QbTest will receive usual care, as outlined above plus the QbTest. The QbTest takes approximately 20 minutes to complete and the results will be discussed with the psychiatrist and the young person, this will take approximately 10 minutes.

Primary Outcomes

- Eligibility rate recorded as the number of eligible young people against the total number of young people identified with ADHD needs;
- Recruitment rate recorded as the number of eligible young people who consent to participate;
- Acceptability of randomisation recorded as the number of young people randomised;
- Acceptability of trial participation recorded as the number of eligible young people who drop out after receiving allocation;
- Attrition rate recorded as the number of young people who consent to participate that remain in the study until the end of follow up at 6 months.

Secondary Outcomes

- Behaviour as measured using the Strengths and Difficulties Questionnaire (SDQ) [36] at baseline, 3 and 6 months. The SDQ is a brief behavioural screening questionnaire. It contains 25 items divided between five scales: emotional symptoms; conduct problems; hyperactivity/inattention; peer relationship problems and prosocial behaviour. It takes 5-10 minutes to complete and is completed with the young person.
- ADHD symptoms are measured using the Brief Barkley ADHD Rating Scale (B-BAARS) [37] at baseline, 3 and 6 months. The B-BAARS is a newly developed short (6 item) screen for ADHD. It takes 5 minutes to complete and is completed with the young person.

For peer review only - http://bmjopen.bmj.com/site/about/guidelines.xhtml

- Health-related quality of life is measured using the Child Health Utility Instrument (CHU-9D) [38] at baseline, 3 and 6 months. It takes 5 minutes to complete and is completed with the young person.
- Objective rating of symptoms and functioning is measured by the Swanson, Nolan and Pelham Questionnaire (SNAP-IV 26) [39] and Children's Global Assessment Scale (C-GAS) [40] at baseline, 3 and 6 months. The SNAP-IV 26 screens for nine symptoms of ADHD hyperactive-impulsive type, nine symptoms of ADHD inattentive type, and eight symptoms of oppositional defiant disorder as defined in the DSM-IV. The SNAP-IV is completed by a teacher within the CYPSE. The C-GAS is a 0-100 scale completed by a clinician which integrates psychological, social and academic functioning.
- Number and duration of (in minutes) consultations/appointments until confirmed ADHD diagnosis as recorded on a pro-forma completed by clinicians after each consultation with the young person.
- Number of days until a confirmed diagnosis is reached using file information.
- Contact with services is measured using the modified Client Service Receipt Inventory (CSRI) [41] at baseline, 3 and 6 months. It takes 5-10 minutes to complete and is completed with the young person.
- Number of reordered behavioural incidents and adjudications are collecting using prison file information at baseline, 3 and 6 months.

Insert Table 1 about here

Sample Size and Recruitment

The required sample is 60 young people, 30 per study arm. This is large enough to test the feasibility of the research procedures and to establish a mean and standard deviation on each outcome measure [42]. Five young people are to be randomised into the study each month. Recruitment rates and the final target will be used to inform the decision to proceed to a definitive trial.

For peer review only

Attrition will be minimised by having robust trial procedures to prevent data loss. Procedures have been developed with our Patient and Public Involvement (PPI) groups and tested for maintaining contact with participants. Recognising that this population can be difficult to follow-up, follow-up data collection points will occur within broad time windows. Having very broad time windows allows for as many follow-ups to be completed within the CYPSE, but also ensuring that no young people are excluded from the study if they miss a follow-up.

Randomisation and Blinding

Randomisation will be achieved by means of concealed random allocation conducted using an online pseudo-random list hosted by Sealedenvelope.com with random permuted blocks of varying sizes. Randomisation will be undertaken by the researcher, who will inform healthcare staff of trial arm allocation.

Given the nature of the intervention, it is not possible to blind participants or those involved in delivering the intervention. It is also not possible to blind the field researcher due to the researcher and staff delivering the intervention working within the confines of a secure environment. It is also not possible to use a second researcher to collect follow-up data (who does not know trial arm allocation) as this is logistically very challenging and ultimately conflicts with the idea of building a strong researcher-participant relationship to enhance follow-up rates. Therefore, to minimise any potential bias, protocolising the collection of outcome measures which will be overseen by the onsite Principal Investigator and trial manager (CL). None of the outcome measures completed by the young person with the researcher present are particularly susceptible to bias as they are standardised, self-complete questionnaires. The researcher may need to support the young person in completing questionnaires due to literacy levels and protocolising collection ensures that support is consistent and unbiased. The study statistician (LAC) will be blind to arm allocation.

Data Collection

For peer review only - http://bmjopen.bmj.com/site/about/guidelines.xhtml
For peer review only

Baseline data collection (t1)

The researcher will deliver the baseline data collection interview using a narrative conversational format. Demographics and questions from other measures are incorporated into a specially constructed flexible paper Case Report Form which avoids duplication of subject matter in order to reduce disengagement or irritability.

Follow-Up (t2-t3)

If the young person is to be released during the study, the researcher will meet with them usually within the week prior to release. During this meeting, the researcher will confirm the contact information provided at baseline and make any amendments to the information (e.g. change of phone number). Once discharged the researcher will contact the participant again in the community, to maintain engagement, confirm contact details and arrange the follow-up in the community.

The 3-month follow-up can take place between 61 and 151 days post randomisation, although the researcher will endeavour to complete data collection as close to the 3 month (90 day) point. The 6-month follow-up can take place between 152 and 244 days post randomisation, although the researcher will endeavour to complete data collection close to the 6 month point (182 days).

The follow-up may take place in prison or in the community, depending on if the young person has been discharged. In the community, the researcher will arrange to meet the young person at a convenient location. Where possible, interviews will be conducted in the premises of services that the young person is engaged with in order to minimise risk to the researcher. Where this is not possible, the researcher will arrange to conduct the interviews in a suitable location in the community and adhere to the Local Lone Working Policy.

Process Evaluation

For peer review only - http://bmjopen.bmj.com/site/about/guidelines.xhtml

QbTest in itself may not be defined as a complex intervention requiring a process evaluation to understand the mechanisms of impact e.g. how QbTest produces change, however, delivering an intervention within a criminal justice setting is complex. Understanding the effects of the context as barriers or facilitators is critical for interpreting the findings and generalising beyond it to a full trial. The process evaluation will follow the MRC guidelines [43] and will be conducted in parallel to the trial and will assess:

- the acceptability of randomisation (interviews);
- the acceptability of outcome measures (completeness of each measure and interviews);
- the numbers of young people agreeing to sit the QbTest and the number who complete QbTest;
- feedback from young people receiving the QbTest and health professionals using a questionnaire;
- the acceptability of the QbTest via interviews with young people; and
- health professionals and other CYPSE staff to obtain perspectives on facilitators and barriers to using the QbTest.

Data collection

QbTest completion rates: A record will be taken of the numbers of participants who agree to sit and also complete the QbTest.

QbTest opinion questionnaire: All young people who receive QbTest will be asked to complete the QbTest opinion questionnaire at 3 months. The questionnaire contains 11 questions e.g. ‘the QbTest results were difficult to understand’ and the young person is asked to rate each item on a 5-point scale. At month 15, clinicians involved in the QbTest will be asked to complete the clinician version, which contains 13 questions rated on a 5-point scale and two free text response questions [29].

For peer review only

Interviews with young people: Randomisation: A sub-sample of young people in both trial arms will be interviewed using a semi-structured interview schedule to further explore acceptability of the randomisation process. Young person's age and time of randomisation (beginning or end of study) will be used to purposively select the sample. Outcomes: A sub-sample of young people in both trial arms will be interviewed using a semi-structured interview schedule to further explore acceptability of completion of the outcome measures. Young person's age and time of randomisation (beginning or end of study) will be used to purposively select the sample. QbTest: A sub-sample of young people who receive QbTest will be interviewed using a semi-structured interview schedule to further explore acceptability of the QbTest and its administration as well as study processes and procedures. Scores/responses in the QbTest opinion questionnaire, young person's age and whether or not the QbTest was completed will be used to purposively select the sample. Up to 20 young people will be interviewed.

Interviews with healthcare professionals and CYPSE staff: Healthcare professionals and CYPSE staff will be invited to participate in qualitative interviews to further explore acceptability and feasibility of administering and implementing the QbTest within usual assessment practices and procedures, including the barriers to its use and reasons for non-completion. Scores/responses in the QbTest Opinion Questionnaire will be used to purposively select healthcare professionals. Up to 10 professionals will be interviewed.

Data Analysis and Management

Data analysis will be mainly descriptive [44]. All measures will be summarised by group across follow-up time with mean and standard deviation for normally distributed data, median for skewed variables and frequency (percentage) for categorical data. All statistical analysis will be conducted using Stata v.15. No interim analysis is planned.

Interviews will be conducted and recorded by the researcher using an encrypted Dictaphone. All qualitative data will be analysed using thematic analysis [45] with the aid of NVivo. Data

For peer review only - http://bmjopen.bmj.com/site/about/guidelines.xhtml

will be coded inductively into themes, creating a detailed coding scheme, allowing for the investigation of emergent patterns between individual codes and boarder emergent themes.

Data will be double entered and stored on an encrypted and password protected database at the University of Manchester. Double-entered data will be compared for discrepancies and discrepant data will be verified using the original paper data sheets (between LWC and CL).

Monitoring

Adverse Events

Serious adverse events (SAEs) will be recorded by the research team and reported to the chief investigator and Trial Steering Committee (TSC). Any SAEs deemed to have a causal relationship to trial participation will be reported to the sponsor within 24 hours of the chief investigator being informed.

Trial Management and Oversight

The TSC will oversee the conduct and safety of the trial and includes the role of the data monitoring. The Committee includes an independent chair, independent members, a patient and public representative and the chief investigator. Representatives from both the Sponsor and funding organisations will be invited to study-related elements of the TSC meetings as observers. The TSC will meet quarterly.

Audit

The Trial Coordinator or a nominated designee of the Sponsor shall carry out monitoring of trial data as an ongoing activity. Trial data and evidence of monitoring and audits will be made available for inspection by the Research Ethics Committee as required.

ETHICS and DISSEMINATION

For peer review only

For peer review only - http://bmjopen.bmj.com/site/about/guidelines.xhtml

Health Research Authority approvals have been granted. Only the research team will have access to the study data, which will be stored in secure locked files or password-protected databases. Data will be available for inspection by the ethics committee on request. Changes to the protocol will be communicated to the ethics committee and trial registries by the trial manager (CL).

Consent Process

Obtaining informed consent or assent will be in accordance with ethical guidance and Good Clinical Practice. The researcher will be trained to assess for capacity to consent in young people. This will include assessing if the young person has the ability to understand the information relevant to the decision, retain the information, use or weigh the information as part of the process for decision making and communicate the decision to the researcher.

The researcher will explain what participation in the study involves and how much time will be involved. They will also explain that participation is voluntary, that they can withdraw at any time and at any point and that their decision to participate, or not, will have no adverse effect on the care that they receive or their other legal rights. The researcher will also discuss the arrangements in place to ensure confidentiality (and limits of this) and data protection. Throughout this process, the potential participant will be given an opportunity to ask questions. Potential participants will be made aware of circumstances in which confidentiality would be broken (if they or someone else identified may be at potential risk of harm; for example, self-harm, breaches of security and violence, including acts of terrorism/radicalisation).

Having had the opportunity to discuss their involvement in the study and ask questions about it, potential participants will be asked if they would like time (minimum 24 hours) to consider taking part or if they would like to sign the Consent Form if they are willing to take part.

Patient and Public Involvement (PPI)

For peer review only - http://bmjopen.bmj.com/site/about/guidelines.xhtml

PPI significantly informs and impacts on this project. NB (co-applicant and co-author) is the project PPI representative and is directly involved in the project providing her lived experience expertise throughout the project ensuring that the perspective of young people with ADHD and their parents are considered throughout. The project has PPI representatives from the community and also within the CYPSE and both groups have helped develop the information leaflets for the study and also helped develop and refine research procedures e.g. how best to explain what participation in the study involves and how best to achieve community follow-ups. The CYPSE PPI group meet with the researcher (LWC) on a regular basis to feed into the project. The PPI groups will be involved in the analysis, reporting and dissemination of the research.

Dissemination

The findings from the trial will be used to inform the design, feasibility and acceptability of a future definitive trial. The findings will be published in peer-reviewed journals, presented at relevant conferences and disseminated to the public via summaries co-created with our PPI group.

DISCUSSION

Secure settings are complex environments in which to conduct trials [46] and there is a dearth of trials within the CYPSE. In the context of this challenging environment, a decision will be taken as to whether feasibility and acceptability are adequate for a full multi-centre randomised controlled trial.

Current service provision for ADHD in the CYPSE is ad hoc and uncoordinated. There is growing evidence within community settings [30, 32, 33, 34] that QbTest has the potential to significantly improve the current assessment pathway having a direct impact on young people's health, service delivery and criminal justice outcome.

For peer review only - http://bmjopen.bmj.com/site/about/guidelines.xhtml

Declarations

Ethics approval and consent to participate

This protocol (version 2) was approved by NHS Wales REC 3 (18/WA/0347) 15th February 2019 and HMPPS NRC 2018-315.

Consent for publication

Not applicable

Availability of data and material

Due to the population of research participants involved in this study individual-level data are not publicly available.

Competing interests

The authors declare that they have no competing interests.

Funding

The study is funded by the NIHR RfPB (Grant number: PB-PG-1216-20007).

Disclaimer

The views expressed are those of the author(s) and not necessarily those of the NHS, the NIHR of the Department of Health. The study sponsor and funders have no role in study design, including collection, management, analysis and interpretation of data; writing of the report and the decision to submit the report for publication.

Author Contributions

For peer review only

PC is the chief investigator and has overall responsibility for the design, conduct and decision to submit for publication. The study design was conceived by PC, CL (trial manager) and CH (co-investigator). CL and PC wrote the protocol with approval from all authors. LAC (co-investigator) is the study statistician and designed and wrote the analysis plan. LAC, CH, BB, SY, NB and AK are co-investigators on the NIHR RfPB grant and contributed to the study design, reviewed and commentated on the drafts of the manuscript. LWC & MR are the researchers collecting the trial data. All authors read and approved the final manuscript.

Trial Sponsor

Pennine Care NHS Foundation Trust; Reagan Blyth, Director of Service Modelling, Research and Innovation, Pennine Care NHS Foundation Trust, Trust Headquarters, 225 Old Street, Ashton-under-Lyne, OL6 7SR, reagan.blyth@nhs.net

For peer review only

References

1 Young S, Moss D, Sedgwick O, Fridman M, Hodgkins P. A meta-analysis of the prevalence of attention deficit hyperactivity disorder in incarcerated populations. Psychol Med. 2015; doi: 10.1017/S0033291714000762.

2 Baggio S, Fructuoso A, Guimaraes M, Fois E, Golay D, Heller P, et al. (2018) Prevalence of attention deficit hyperactivity disorder in detention settings: a systematic review and meta-analysis. Front Psychiatry. 2018; doi: 10.3389/fpsyt.2018.00331.

3 Hughes N, Williams H, Chitsabeasan P, Davies R, Mounce L. Nobody made the connection: The prevalence of neurodisability in young people who offend. The Children's Commissioner. 2012. https://www.childrenscommissioner.gov.uk/sites/default/files/publications/Nobody%20made%20the%20connection.pdf Accessed 17 May 2019.

4 Barkley RA. Attention-Deficit Hyperactivity Disorder: A Handbook for Diagnosis and Treatment. 3rd eds. New York: Guildford Press; 2006

5 Shaw M, Hodgkins P, Caci H, Young S, Kahle J, Wood AG et al. A systematic review and analysis of long-term outcomes in attention deficit hyperactivity disorder: effects of treatment and non-treatment. BMC Medicine. 2012; doi: 10.1186/1741-7015-10-99.

6 National Institute for Health and Care Excellence. Attention deficit hyperactivity disorder: diagnosis and management (CG72). National Institute for Health and Care Excellence. 2008. https://www.nice.org.uk/guidance/cg72?unlid=52068493320161144181 4 Accessed 17 May 2019.

7 National Institute for Health and Care Excellence. Attention deficit hyperactivity disorder (QS39). National Institute for Health and Care Excellence. 2013. https://www.nice.org.uk/guidance/qs39 Accessed 17 May 2019.

8 Takeda T, Ambrosini P, deBerardinis R, Elia J. What can ADHD without comorbidity teach us about comorbidity? Res Dev Disabil. 2012; doi: 10.1016/j.ridd.2011.09.024.

For peer review only

For peer review only - http://bmjopen.bmj.com/site/about/guidelines.xhtml

9 Moffitt TE. Juvenile delinquency and Attention Deficit Disorder: Developmental trajectories from age 3 to 15. Child Dev. 1990; 61: 893-910.

10 Moffitt TE. (1993). Adolescence-limited and life-course-persistent antisocial behavior: A developmental taxonomy. Psychol Rev. 1993; 100: 674-701.

11 Eme R. Attention deficit hyperactivity disorder and the family court. Fam Court Rev. 2009; doi: 10.1111/j.1744-1617.2009.01279.x.

12 Mental Health Taskforce. The five year forward view of mental health: A report from the independent Mental Health Taskforce to the NHS in England. 2016 https://www.england.nhs.uk/wpcontent/uploads/2016/02/Mental-Health-Taskforce-FYFV-final.pdf Accessed 17 May 2019.

13 Young S, Sedgwick O, Fridman M, Gudjonsson G, Hodgkins P, Lantigua M, et al. Comorbid psychiatric disorders amongst incarcerated ADHD populations: A meta-analysis. Psychol Med. 2015 doi: 10.1017/S0033291715000598.

14 Biederman J, Petty C, Clarke A, Lomedico A, Faraone SV. Predictors of persistent ADHD: An 11-year follow-up study. J Psychiatr Res. 2011; doi: 10.1016/j.jpsychires.2010.06.009.

15 Arnold LE, Hodgkins P, Kahle J, Madhoo M, Kewley G. Long-term outcomes of ADHD: Academic achievement and performance. J Atten Disord. 2015; pii: 1087054714566076.

16 D'Amico F, Knapp M, Beecham J, Sandberg S, Taylor E, et al. Use of services and associated costs for young adults with childhood hyperactivity/conduct problems: 20-year follow-up. Br J Psychiatry. 2014; doi: 10.1192/bjp.bp.113.131367.

17 Khong B.The lifetime costs of attention deficit hyperactivity disorder (ADHD). Centre for Mental Health. 2014. https://www.centreformentalhealth.org.uk/costs-of-adhd Accessed 17 May 2019.

18 Young S, Gonzalez R, Fridman M, Hodgkins P, Kim K, Gudjonsson G. The economic consequences of ADHD in the Scottish prison system. BMC Psychiatry. 2018. doi: 10.1186/s12888-018-1792-x.

For peer review only

19 Young S, González R, Gudjonsson GH. Health-related quality of life in prisoners with attention deficit/hyperactivity disorder and head injury. BMC Psychiatry. 2018; doi: 10.1186/s12888-018-1785-9.

20 Royal College of Paediatrics and Child Health. Healthcare standards for children and young people in secure settings. Royal College of Paediatrics and Child Health. 2013 https://www.rcpch.ac.uk/sites/default/files/2018-03/healthcare_standards_for_children_and_young_people_in_secure_settings_june_2013.pdf Accessed 17 May 2019.

21 Chitsabesan P, Lennox C, Theodosiou L, Law H, Bailey S, Shaw J. The development of the comprehensive health assessment tool for young offenders within the secure estate. J Forens Psychiatry Psychol. 2014; doi: 10.1080/14789949.2014.882387

22 Ministry of Justice. Youth justice annual statistics: 2014 to 2015. Ministry of Justice. 2016 https://www.gov.uk/government/statistics/youth-justice-annual-statistics-2014-to-2015 Accessed 17 May 2019.

23 Ministry of Justice. What works in managing young people who offend? Summary of the international evidence. Ministry of Justice. 2016. https://www.gov.uk/government/publications/whatworksin-managing-young-people-who-offend Accessed 17 May 2019.

24 Young S, Gudjonsson GH, Wells J, Asherson P, Theobald D, et al. Attention deficit hyperactivity disorder and critical incidents in a Scottish prison population. Pers Individ Dif. 2009; doi: 10.1016/j.paid.2008.10.003.

25 Young S, Misch P, Collins P, Gudjonsson GH. Predictors of institutional behavioural disturbance and offending in the community among young offenders. J Forens Psychiatry Psychol. 2011; doi: 10.1080/14789949.2010.495991.

26 Ministry of Justice. Safety in Custody Statistics Bulletin: Deaths in prison custody to September 2016; Assaults and self-harm to June 2016. Ministry of Justice. 2016. https://www.gov.uk/government/uploads/system/uploads/attachment_data/file/562897/safety-incustodybulletin.pdf Accessed 17 May 2019.

For peer review only - http://bmjopen.bmj.com/site/about/guidelines.xhtml

27 Lichtenstein P, Halldner L, Zetterqvist J, Sjolander A, Serlachius E, Fazel S, et al. Medication for attention deficit-hyperactivity disorder and criminality. N Engl J Med. 2012; doi: 10.1056/NEJMoa1203241.

28 Reh V, Schmidt M, Lam L, Schimmelmann BG, Hebebrand J, Rief W, et al. Behavioral assessment of core ADHD symptoms using the QbTest. J Atten Disord. 2015; doi: 10.1177/1087054712472981.

29 Reh V, Schidt M, Rief W, Christiansen H. Preliminary evidence for altered motion tracking-based hyperactivity in ADHD siblings. Behavl Brain Funct. 2014; doi: 10.1186/1744-9081-10-7.

30 Hall CL, Valentine AZ, Groom MJ, Walker GM, Daley D, Hollis C. The clinical utility of the continuous performance test and objective measures of activity for diagnosing and monitoring ADHD in children: a systematic review. Eur Child Adolesc Psychiatry. 2016. doi: 10.1007/s00787-015-0798-x.

31 Vogt C, Shameli A. Assessments for attention-deficit hyperactivity disorder: use of objective measurements. Psychiatrist. 2011; doi: 10.1192/pb.bp.110.032144

32 Hall C, Selby K, Boliang G, Althea V, Walker G, Hollis C. Innovations in Practice: an objective measure of attention, impulsivity and activity reduces time to confirm attention deficit/hyperactivity disorder diagnosis in children – a completed audit cycle. Child Adolesc Ment Health. 2016; doi: 10.1111/camh.12140

33 Hollis C, Hall CL, Guo B, James M, Boadu J, Groom M, et al. The impact of a computerised test of attention and activity (QbTest) on diagnostic decision-making in children and young people with suspected attention deficit hyperactivity disorder: single-blind randomised controlled trial. J Child Psychol Psychiatry. 2018; doi:10.1111/jcpp.1292.1

34 Hall C, Valentine AZ, Walker GM, Ball HM, Cogger H, Daley D, et al. Study of user experience of an objectice test (QbTest) to aid ADHD assessement and medication management: a multi-methods approach. BMC Psychiatry. 2017; doi: 10.1186/s12888-017-1222-5.

For peer review only

35 Medical Research Council. Developing and evaluating complex interventions: new guidance. Medical Research Council. 2008. http://www.mrc.ac.uk/documents/pdf/complexinterventionsguidance/ Accessed 17 May 2019.

36 Goodman R. The Strengths and Difficulties Questionnaire: A research note. J Child Psychol Psychiatry. 1997; 38: 581–586

37 Young S, González RA, Mutch L, Mallet-Lambert I, O'Rourke L. et al. Diagnostic accuracy of a brief screening tool for attention deficit hyperactivity disorder in UK prison inmates. Psychol Med. 2016; doi: 10.1017/S0033291716000039.

38 Stevens KJ. Assessing the performance of a new generic measure of health related quality of life for children and refining it for use in health state valuation. Appl Health Econ Health Policy. 2011; doi: 10.2165/11587350-000000000-00000.

39 Swanson JM, Kraemer HC, Hinshaw S P, Arnold LE, Conners CK, Abikokk HB, et al. Clinical relevance of the primary findings of the MTA: success rates based on severity of ADHD and ODD symptoms at the end of treatment. J Am Acad Child Adolesc Psychiatry. 2001; 40: 168-79.

40 Shaffer N, Gould MS, Brasic J. A children's Global Assessment Scale (CGAS). Arch Gen Psychiatry. 1983; 40:1228-1231.

41 Beecham J, Knapp M. Costing psychiatric interventions. In G. Thornicroft (ed.) Measuring Mental Health Needs, Gaskell, 2nd edition, p200-224. 2001.

42 Hertzog MA. Considerations in determining sample size for pilot studies. Res Nurs Health. 2008; doi: 10.1002/nur.20247.

43 Moore G, Audrey S, Barker M, Bond L, Bonell C. Hardman W, et al. Process evaluation of complex interventions: Medical Research Council guidance. BMJ. 2015; doi: 10.1136/bmj.h1258.

44 Lancaster GA, Dodd SR, Williamson PR: Design and analysis of pilot studies: recommendations for good practice. J Eval Clin Pract. 2004, doi: 10.1111/j..2002.384.doc.x.

For peer review only - http://bmjopen.bmj.com/site/about/guidelines.xhtml

45 Braun V, Clarke V. Using thematic analysis in psychology. Qual Res Psychol. 2006; 3: 77-101.

46 Pettus-Davis C, Howard MO, Dunningan A, Scheyett AM, Roberts-Lewis A. Using randomized controlled trial to evaluate interventions for releasing prisoners. Res Soc Work Pract. 2015; doi: 10.1177/1049731515579203

Table 1: Summary of study schedule

		Screening	Baseline	Allocation	Follow-Up	
**TIMEPOINT**		*t0*	*t1*		*+3 mth t2*	*+6 mth t3*
**ENROLMENT:**						
**Eligibility screen**		X				
**Informed consent**		X				
Parental consent (< 16)		X				
**Allocation**				X		
**INTERVENTIONS:**						
Intervention Group:	***QbTest***			↔		
	Usual care	↔	↔	↔	↔	↔
Control Group:	***Usual care***	↔	↔	↔	↔	↔
**ASSESSMENTS:**						
Demographics			X		X	X
SDQ			X		X	X
B-BAARS			X		X	X
CHU-9D			X		X	X
SNAP-IV			X		X	X
C-GAS			X		X	X
ADHD Diagnosis & Clinical Confidence					X	X
Health Records			X		X	X
Prison Records			X		X	X
Modified CSRI			X		X	X
Contact Sheet			X		X	
**SAFETY MONITORING:**						
Adverse event reporting		↔	↔	↔	↔	↔

SDQ = Strengths and Difficulties Questionnaire; B-BAARS = Brief Barkley ADHD Rating Scale; CHU-9D = Child Health Utility Instrument; SNAP-IV = Swanson, Nolan and Pelham Questionnaire; C-GAS = Children's Global Assessment Scale; CSRI = Client Service Receipt Inventory

For peer review only - http://bmjopen.bmj.com/site/about/guidelines.xhtml

SPIRIT
STANDARD PROTOCOL ITEMS: RECOMMENDATIONS FOR INTERVENTIONAL TRIALS

SPIRIT 2013 Checklist: Recommended items to address in a clinical trial protocol and related documents*

Section/item	Item No	Description	Addressed on page number
**Administrative information**			
Title	1	Descriptive title identifying the study design, population, interventions, and, if applicable, trial acronym	________1_____
Trial registration	2a	Trial identifier and registry name. If not yet registered, name of intended registry	________3_____
	2b	All items from the World Health Organization Trial Registration Data Set	________n/a___
Protocol version	3	Date and version identifier	_______18_____
Funding	4	Sources and types of financial, material, and other support	_______18_____
Roles and responsibilities	5a	Names, affiliations, and roles of protocol contributors	_______18_____
	5b	Name and contact information for the trial sponsor	_______19_____
	5c	Role of study sponsor and funders, if any, in study design; collection, management, analysis, and interpretation of data; writing of the report; and the decision to submit the report for publication, including whether they will have ultimate authority over any of these activities	_______18_____
	5d	Composition, roles, and responsibilities of the coordinating centre, steering committee, endpoint adjudication committee, data management team, and other individuals or groups overseeing the trial, if applicable (see Item 21a for data monitoring committee)	_______15__

For peer review only

**Introduction**			
Background and rationale	6a	Description of research question and justification for undertaking the trial, including summary of relevant studies (published and unpublished) examining benefits and harms for each intervention	___4-6 ______
	6b	Explanation for choice of comparators	_____n/a_____
Objectives	7	Specific objectives or hypotheses	______7______
Trial design	8	Description of trial design including type of trial (eg, parallel group, crossover, factorial, single group), allocation ratio, and framework (eg, superiority, equivalence, noninferiority, exploratory)	_____7_______
**Methods: Participants, interventions, and outcomes**			
Study setting	9	Description of study settings (eg, community clinic, academic hospital) and list of countries where data will be collected. Reference to where list of study sites can be obtained	______7______
Eligibility criteria	10	Inclusion and exclusion criteria for participants. If applicable, eligibility criteria for study centres and individuals who will perform the interventions (eg, surgeons, psychotherapists)	______7______
Interventions	11a	Interventions for each group with sufficient detail to allow replication, including how and when they will be administered	________8_____
	11b	Criteria for discontinuing or modifying allocated interventions for a given trial participant (eg, drug dose change in response to harms, participant request, or improving/worsening disease)	_____n/a_____
	11c	Strategies to improve adherence to intervention protocols, and any procedures for monitoring adherence (eg, drug tablet return, laboratory tests)	______n/a____
	11d	Relevant concomitant care and interventions that are permitted or prohibited during the trial	________n/a___
Outcomes	12	Primary, secondary, and other outcomes, including the specific measurement variable (eg, systolic blood pressure), analysis metric (eg, change from baseline, final value, time to event), method of aggregation (eg, median, proportion), and time point for each outcome. Explanation of the clinical relevance of chosen efficacy and harm outcomes is strongly recommended	______9-10___
Participant timeline	13	Time schedule of enrolment, interventions (including any run-ins and washouts), assessments, and visits for participants. A schematic diagram is highly recommended (see Figure)	_____Figure 1__

For peer review only - http://bmjopen.bmj.com/site/about/guidelines.xhtml

Sample size	14	Estimated number of participants needed to achieve study objectives and how it was determined, including clinical and statistical assumptions supporting any sample size calculations	_____10-11____
Recruitment	15	Strategies for achieving adequate participant enrolment to reach target sample size	______10-11___

Methods: Assignment of interventions (for controlled trials)

Allocation:

Sequence generation	16a	Method of generating the allocation sequence (eg, computer-generated random numbers), and list of any factors for stratification. To reduce predictability of a random sequence, details of any planned restriction (eg, blocking) should be provided in a separate document that is unavailable to those who enrol participants or assign interventions	_____11______
Allocation concealment mechanism	16b	Mechanism of implementing the allocation sequence (eg, central telephone; sequentially numbered, opaque, sealed envelopes), describing any steps to conceal the sequence until interventions are assigned	______11_____
Implementation	16c	Who will generate the allocation sequence, who will enrol participants, and who will assign participants to interventions	______11_____
Blinding (masking)	17a	Who will be blinded after assignment to interventions (eg, trial participants, care providers, outcome assessors, data analysts), and how	______11__
	17b	If blinded, circumstances under which unblinding is permissible, and procedure for revealing a participant's allocated intervention during the trial	____11___

Methods: Data collection, management, and analysis

Data collection methods	18a	Plans for assessment and collection of outcome, baseline, and other trial data, including any related processes to promote data quality (eg, duplicate measurements, training of assessors) and a description of study instruments (eg, questionnaires, laboratory tests) along with their reliability and validity, if known. Reference to where data collection forms can be found, if not in the protocol	____11-12_____
	18b	Plans to promote participant retention and complete follow-up, including list of any outcome data to be collected for participants who discontinue or deviate from intervention protocols	____11-12_____

Data management	19	Plans for data entry, coding, security, and storage, including any related processes to promote data quality (eg, double data entry; range checks for data values). Reference to where details of data management procedures can be found, if not in the protocol	_______14_____
Statistical methods	20a	Statistical methods for analysing primary and secondary outcomes. Reference to where other details of the statistical analysis plan can be found, if not in the protocol	_______14_____
	20b	Methods for any additional analyses (eg, subgroup and adjusted analyses)	______n/a______
	20c	Definition of analysis population relating to protocol non-adherence (eg, as randomised analysis), and any statistical methods to handle missing data (eg, multiple imputation)	______n/a_____
**Methods: Monitoring**			
Data monitoring	21a	Composition of data monitoring committee (DMC); summary of its role and reporting structure; statement of whether it is independent from the sponsor and competing interests; and reference to where further details about its charter can be found, if not in the protocol. Alternatively, an explanation of why a DMC is not needed	______15___
	21b	Description of any interim analyses and stopping guidelines, including who will have access to these interim results and make the final decision to terminate the trial	______n/a____
Harms	22	Plans for collecting, assessing, reporting, and managing solicited and spontaneously reported adverse events and other unintended effects of trial interventions or trial conduct	_______15_____
Auditing	23	Frequency and procedures for auditing trial conduct, if any, and whether the process will be independent from investigators and the sponsor	_______15_____
**Ethics and dissemination**			
Research ethics approval	24	Plans for seeking research ethics committee/institutional review board (REC/IRB) approval	______15 & 18__
Protocol amendments	25	Plans for communicating important protocol modifications (eg, changes to eligibility criteria, outcomes, analyses) to relevant parties (eg, investigators, REC/IRBs, trial participants, trial registries, journals, regulators)	______15______

For peer review only - http://bmjopen.bmj.com/site/about/guidelines.xhtml

Consent or assent	26a	Who will obtain informed consent or assent from potential trial participants or authorised surrogates, and how (see Item 32)	______16____
	26b	Additional consent provisions for collection and use of participant data and biological specimens in ancillary studies, if applicable	_______n/a____
Confidentiality	27	How personal information about potential and enrolled participants will be collected, shared, and maintained in order to protect confidentiality before, during, and after the trial	______16___
Declaration of interests	28	Financial and other competing interests for principal investigators for the overall trial and each study site	______18______
Access to data	29	Statement of who will have access to the final trial dataset, and disclosure of contractual agreements that limit such access for investigators	_______18____
Ancillary and post-trial care	30	Provisions, if any, for ancillary and post-trial care, and for compensation to those who suffer harm from trial participation	______n/a_____
Dissemination policy	31a	Plans for investigators and sponsor to communicate trial results to participants, healthcare professionals, the public, and other relevant groups (eg, via publication, reporting in results databases, or other data sharing arrangements), including any publication restrictions	______17
	31b	Authorship eligibility guidelines and any intended use of professional writers	______n/a______
	31c	Plans, if any, for granting public access to the full protocol, participant-level dataset, and statistical code	_______18_____
**Appendices**			
Informed consent materials	32	Model consent form and other related documentation given to participants and authorised surrogates	_______n/a____
Biological specimens	33	Plans for collection, laboratory evaluation, and storage of biological specimens for genetic or molecular analysis in the current trial and for future use in ancillary studies, if applicable	_______n/a____

*It is strongly recommended that this checklist be read in conjunction with the SPIRIT 2013 Explanation & Elaboration for important clarification on the items. Amendments to the protocol should be tracked and dated. The SPIRIT checklist is copyrighted by the SPIRIT Group under the Creative Commons "Attribution-NonCommercial-NoDerivs 3.0 Unported" license.

For peer review only - http://bmjopen.bmj.com/site/about/guidelines.xhtml

FACT: A randomised controlled trial to assess the feasibility of QbTest in the assessment process of attention deficit hyperactivity disorder (ADHD) for young people in prison: A feasibility trial protocol.

Journal:	*BMJ Open*
Manuscript ID	bmjopen-2019-035519.R1
Article Type:	Protocol
Date Submitted by the Author:	27-Nov-2019
Complete List of Authors:	Lennox, Charlotte; The University of Manchester, Division of Psychology and Mental Health<br>Hall, Charlotte; University of Nottingham, Division of Psychiatry & Applied Psychology, Institute of Mental Health,<br>Carter, Lesley-Anne; The University of Manchester, Centre for Biostatistics, Division of Population Health, Health Services Research and Primary Care<br>Beresford, Bryony; University of York, Social Policy Research Unit<br>Young, Susan; Psychology Services Limited<br>Kraam, Abdullah ; Rotherham Doncaster and South Humber Mental Health NHS Foundation Trust<br>Brown, Nikki; University of Nottingham<br>Wilkinson-Cunningham, Lloyd; Leeds Community Healthcare NHS Trust,<br>Reeves, Mindy; The University of Manchester<br>Chitsabesan, Prathiba; Pennine Care NHS Foundation Trust
**Primary Subject Heading**:	Mental health
Secondary Subject Heading:	Diagnostics
Keywords:	Child & adolescent psychiatry < PSYCHIATRY, Forensic psychiatry < PSYCHIATRY, Clinical trials < THERAPEUTICS, QUALITATIVE RESEARCH

SCHOLARONE™
Manuscripts

FACT: A randomised controlled trial to assess the feasibility of QbTest in the assessment process of attention deficit hyperactivity disorder (ADHD) for young people in prison: A feasibility trial protocol.

C Lennox[1]; C L Hall; L A Carter; B Beresford; S Young; A Kraam; N Brown; L Wilkinson-Cunningham; M Reeves; P Chitsabesan

[1]Dr Charlotte Lennox (Corresponding author); Division of Psychology and Mental Health, School of Health Sciences, Room 2.315 Jean McFarlane Building, The University of Manchester, Oxford Road, Manchester, M13 9PL charlotte.lennox@manchester.ac.uk

Dr Charlotte L Hall, Division of Psychiatry & Applied Psychology, Institute of Mental Health, University of Nottingham Innovation Park, Triumph Road, Nottingham, NG7 2TU, charlotte.hall@nottingham.ac.uk

Dr Lesley-Anne Carter, Centre for Biostatistics, Division of Population Health, Health Services Research and Primary Care, University of Manchester, 1.304 Jean McFarlane Building, University of Manchester, Oxford Road, Manchester, M13 9PL lesley-anne.carter@manchester.ac.uk

Professor Bryony Beresford, Social Policy Research Unit, University of York, Heslington, York, YO10 5DD, bryony.beresford@york.ac.uk

Professor Susan Young, Psychology Services Limited, London, UK suzyyoung@aol.com

Dr Abdullah Kraam, Leeds Community Healthcare, HMPYOI Wetherby, York Rd, Wetherby LS22 5ED, a.kraam@nhs.net

Nikki Brown, Patient and Public Involvement (PPI) Representative, nikki.l.brown@sky.com

For peer review only

Lloyd Wilkinson-Cunningham, Leeds Community Healthcare, HMPYOI Wetherby, York Rd, Wetherby LS22 5ED, lloyd.wilkinson-cunningham@nhs.net

Dr Mindy Reeves, Specialty Trainee Doctor in Forensic Child and Adolescent Psychiatry, University of Manchester, mindy.reeves@doctors.org.uk

Dr Prathiba Chitsabesan, Pennine Care NHS Foundation Trust, 225 Old St, Ashton-under-Lyne OL6 7SR, pchitsabesan@nhs.net

For peer review only

For peer review only - http://bmjopen.bmj.com/site/about/guidelines.xhtml

ABSTRACT

Introduction: The prevalence of attention deficit hyperactivity disorder (ADHD) within the Children and Young People Secure Estate (CYPSE) is much higher than seen in the general population. To make a diagnosis of ADHD clinicians draw on information from multiple sources, including parents and teachers. However, obtaining these is particularly difficult for young people in the secure estate. There is increasing evidence in the community that QbTest is able to assist in the accurate and earlier diagnosis of ADHD. The objective of this study is assess the feasibility and acceptability of QbTest in the assessment of ADHD within the CYPSE.

Methods and Analysis: Single centre parallel group feasibility randomised controlled trial. Sixty young people within the CYPSE identified as displaying possible symptoms of ADHD will be randomised to the intervention arm (n = 30; QbTest plus usual care) or control arm (n = 30; usual care). Primary analyses will be descriptive and a process evaluation will be conducted to assess the contexts involved in implementing the intervention. Interviews will be conducted to explore acceptability and thematic analysis will be used to analyse the data.

Ethics and Dissemination: This study was approved by NHS Wales REC 3 (18/WA/0347) on 15th February 2019. The findings will be published in peer-reviewed journals, presented at relevant conferences and disseminated to the public via summaries co-created with our Patient and Public Involvement group.

Trial registration: ISRCTN registry ISRCTN17402196

For peer review only

For peer review only - http://bmjopen.bmj.com/site/about/guidelines.xhtml

Article Summary

Strengths and limitations of this study

- This trial is novel at it is one of the first trials within the Children and Young People Secure Estate (CYPSE).
- A process evaluation will understand the effects of context as barriers or facilitators is critical for interpreting the findings.
- The study will engage meaningfully and substantively with a patient and public involvement group to ensure the research and intervention are both feasible and acceptable.
- This is a feasibility trial and is not powered to show effectiveness of the intervention.
- This feasibility trial is only being conducted in one site.

Keywords: Child & adolescent psychiatry, Forensic psychiatry, Clinical trials, Qualitative research

Word Count: 3990

For peer review only - http://bmjopen.bmj.com/site/about/guidelines.xhtml

INTRODUCTION

In England children and young people aged 10-17 years old, remanded or sentenced into custody are placed in a variety of different secure establishments, known collectively as the Children and Young People Secure Estate (CYPSE). The CYPSE includes: Secure Children's Homes (SCHs), Secure Training Centres (STCs) and Young Offenders Institutions (YOIs). The majority of young people are placed within the YOIs and there are four establishments in England accepting 15–17 year olds.

The prevalence rate of attention deficit hyperactivity disorder (ADHD) in the CYPSE is approximately 30-40%, which is significantly greater than the rate of 5%, seen in the general population [1, 2]. However, a report raised significant concerns about undetected neurodisability, including ADHD within the CYPSE, due to a lack of appropriate screening and assessment processes and training of staff [3]. Additionally, many young people have co-morbid mental health needs contributing to complexity in the assessment process. ADHD is associated with a range of poor outcomes, including a greater risk of developing other mental and physical health needs, educational and occupational problems and offending [4-13]. The social and economic burden of untreated ADHD on society is significant [14-19].

The publication of the Healthcare Standards for Children and Young People in Secure Settings [20] attempted to provide a more standardised approach to the assessment and care provided within the CYPSE. One way was to recommend the use of the Comprehensive Health Assessment Tool (CHAT) [21]. The CHAT was introduced into the CYPSE in 2013 and is

For peer review only - http://bmjopen.bmj.com/site/about/guidelines.xhtml

completed within all CYPSE sites in England, UK. The CHAT is a semi-structured assessment of health needs, delivered by a nurse during the first 10 days of admission. If a nurse from talking with the young person and from observing them believes that they may have a need around difficulty concentrating, restlessness, fidgeting etc. then the nurse would recommend in the CHAT care plan that the young person be referred for a full ADHD assessment.

For a formal clinical diagnosis of ADHD, a key aspect is the use of information from informants such as parents, teachers and health professionals [6-7]. However, access to such information can be difficult to obtain and take a long time to receive, resulting in delays to receiving a confirmed diagnosis. The number of days young people spend in the CYPSE is relatively short [22]. Therefore, informant information is very often not received before the young person leaves and returns back to the community. There is also evidence from a recent review within the CYPSE that establishments find it difficult to provide interventions to young people who are sentenced to fewer than six months, due to delays in assessment and/or diagnosis [23]. All these factors can lead to delays in young people with ADHD receiving a diagnosis and accessing evidence based treatment.

The impact of missed opportunities in diagnosis and accessing appropriate treatment can be significant. Young people with untreated ADHD in the CYPSE have been found to have an eight fold increased frequency of aggressive incidents in secure settings [24, 25]. Reducing violence and aggression is a key priority for Her Majesty's Prison & Probation Service (HMPPS) and Ministry of Justice, given the increasing rates within prisons [26]. Treatment of adult offenders with ADHD using medication has demonstrated a reduction in re-offending of up to 41% [27]. Therefore, there are also likely social and economic benefits to improving the diagnosis and access to treatment for young people with ADHD in the CYPSE.

QbTest (Qbtech Ltd) is a computer task that measures three core aspects of ADHD: attention, impulsivity and motor activity. Performance on the task provides information (via an immediate report) on the three symptom domains of ADHD and a 'summary score' based on deviation

For peer review only - http://bmjopen.bmj.com/site/about/guidelines.xhtml

from a normative data set based on age group and gender. QbTest can be used with individuals with mild learning disabilities as instructions on how to complete the task are visual. Practitioners use the information from the QbTest report in conjunction with the clinical information (patient history, observation and ADHD symptom questionnaire) to inform their decision whether the young person has ADHD or not.

QbTest demonstrates good psychometric properties [28, 29] and has the additional benefit over other computer tasks of measuring activity level, a core symptom of ADHD [30]. There are several studies that demonstrate the clinical utility of QbTest in aiding diagnosis [31, 32]. A recent randomised controlled trial found clinicians with access to the QbTest report were more likely to reach a diagnostic decision about ADHD. At 6-months, 76% of those with a QbTest report had received a diagnostic decision, compared with 50% without a QbTest report. QbTest reduced appointment length, increased clinicians' confidence in their diagnostic decisions and doubled the likelihood of excluding ADHD [33].

The use of the QbTest in the community looks promising [34]. However, there are a number of unknowns in relation to its use in the CYPSE. Implementing new approaches can require more planning due to the regime; therefore the potential barriers and facilitators to implementation need to be understood. The young people within the CYPSE are more complex and have greater co-morbidity than young people accessing community Child and Adolescent Mental Health Services. The acceptability of the QbTest needs to be assessed to see if the same positive findings can be replicated. Primary outcomes in previous QbTest trials [33] have been time to diagnosis, but given the comparative short stay in the CYPSE and difficulty obtaining this data once they leave, it is unclear how reliably this data can be collected and/or if other outcomes, such as in aggressive incidents are more important in this population.

The longer term aim is to undertake a definitive randomised controlled trial of the effectiveness of QbTest in the assessment of ADHD in the CYPSE. An evidence base within the CYPSE needs to be developed in order to change policy and implement the QbTest across the whole

For peer review only

For peer review only - http://bmjopen.bmj.com/site/about/guidelines.xhtml

system. In line with the Medical Research Council guidelines on developing complex interventions and as randomised controlled trials within the CYPSE are scarce, a feasibility study is required [35].

Trial Objectives

To conduct a pragmatic feasibility randomised controlled trial to assess the feasibility and acceptability of QbTest in the assessment of ADHD within the CYPSE.

Trial Design

A parallel two group randomised controlled trial with 1:1 individual participant allocation to QbTest plus usual care (n = 30; intervention group) or usual care alone (n = 30; control group) with a parallel process evaluation. Recruitment started in March 2019 and is due to last 12 months.

For peer review only - http://bmjopen.bmj.com/site/about/guidelines.xhtml

METHODS and ANALYSIS

Setting

The study will be conducted in one YOI site within the CYPSE, in England.

Eligibility Criteria

Inclusion criteria:

- Identified as presenting with ADHD symptoms via the CHAT; and
- Aged 15-18 years old.

Exclusion criteria:

- On remand (uncertain/sudden release);
- Non-English speaking (for pragmatic reasons, but will be included in the main trial testing applicability more widely);
- Previous or current confirmed diagnosis of ADHD;
- Currently receiving ADHD medication;
- Deemed to present risk to either the researcher or the staff;
- Unable to provide informed consent (over 16); and
- Parental/legal guardian consent not received (under 16)

Potential participants will be identified by the prison healthcare team. The CHAT will identify potential participants displaying possible symptoms of ADHD e.g. any ADHD symptom codes

For peer review only

For peer review only - http://bmjopen.bmj.com/site/about/guidelines.xhtml

in CHAT. Sentence status is accessible to healthcare staff via SystmOne[1] and young people on remand will be excluded. Healthcare records will also be searched for previous or current confirmed diagnosis of ADHD and/or currently receiving ADHD medication and those young people will be excluded. The risk presented by potential participants will be assessed by the healthcare team, based on knowledge of the young person, information contained on SystmOne and discussion with prison staff and will take account of the likely risk in prison and community settings. Potential participants whose risk cannot be managed safely will be excluded.

Usual Care

The CHAT is a semi-structured assessment of health needs. It is delivered by a nurse during the first 10 days of admission and includes assessing ADHD needs (not diagnostic) based on clinical history and observation of the young person. Young people identified with potential ADHD on the CHAT are discussed with the Psychiatrist at the Multi-Disciplinary Team. ADHD questionnaires (self, teacher and parent) are completed and reviewed. If further ADHD assessment is required, this includes clinical assessment (history and observation) and obtaining informant information including developmental history (completed by nurse and psychiatrist). This typically involves a minimum of three appointments with the young person and liaison with teachers/parents.

Intervention – QbTest plus usual care

Once the young person has been referred to the Multi-Disciplinary Team the young person is booked into the QbTest clinic. The healthcare team will conduct the QbTest and use the information from the QbTest report in conjunction with the clinical information (patient history, observation and ADHD symptom questionnaire) to inform their decision whether the young person has ADHD or not. Young people randomised to the QbTest will receive usual care, as outlined above plus the QbTest. The QbTest takes approximately 20 minutes to complete and

[1] SystmOne is the clinical record system used within the CYPSE

For peer review only - http://bmjopen.bmj.com/site/about/guidelines.xhtml
For peer review only

the results will be discussed with the psychiatrist and the young person, this will take approximately 10 minutes.

Primary Outcomes

- Eligibility rate recorded as the number of eligible young people against the total number of young people identified with ADHD needs;
- Recruitment rate recorded as the number of eligible young people who consent to participate;
- Acceptability of randomisation recorded as the number of young people randomised;
- Acceptability of trial participation recorded as the number of eligible young people who drop out after receiving allocation;
- Attrition rate recorded as the number of young people who consent to participate that remain in the study until the end of follow up at 6 months.

Secondary Outcomes

- Behaviour as measured using the Strengths and Difficulties Questionnaire (SDQ) [36] at baseline, 3 and 6 months. The SDQ is a brief behavioural screening questionnaire. It contains 25 items divided between five scales: emotional symptoms; conduct problems; hyperactivity/inattention; peer relationship problems and prosocial behaviour. It takes 5-10 minutes to complete and is completed with the young person.
- ADHD symptoms are measured using the Brief Barkley ADHD Rating Scale (B-BAARS) [37] at baseline, 3 and 6 months. The B-BAARS is a newly developed short (6 item) screen for ADHD. It takes 5 minutes to complete and is completed with the young person.
- Health-related quality of life is measured using the Child Health Utility Instrument (CHU-9D) [38] at baseline, 3 and 6 months. It takes 5 minutes to complete and is completed with the young person.
- Objective rating of symptoms and functioning is measured by the Swanson, Nolan and Pelham Questionnaire (SNAP-IV 26) [39] and Children's Global Assessment Scale (C-

For peer review only

For peer review only - http://bmjopen.bmj.com/site/about/guidelines.xhtml

GAS) [40] at baseline, 3 and 6 months. The SNAP-IV 26 screens for nine symptoms of ADHD hyperactive-impulsive type, nine symptoms of ADHD inattentive type, and eight symptoms of oppositional defiant disorder as defined in the DSM-IV. The SNAP-IV is completed by a teacher within the CYPSE. The C-GAS is a 0-100 scale completed by a clinician which integrates psychological, social and academic functioning.

- Number and duration of (in minutes) consultations/appointments until confirmed ADHD diagnosis as recorded on a pro-forma completed by clinicians after each consultation with the young person.
- Number of days until a confirmed diagnosis is reached using file information.
- Contact with services is measured using the modified Client Service Receipt Inventory (CSRI) [41] at baseline, 3 and 6 months. It takes 5-10 minutes to complete and is completed with the young person.
- Number of reordered behavioural incidents and adjudications are collecting using prison file information at baseline, 3 and 6 months.

Sample Size and Recruitment

The required sample is 60 young people, 30 per study arm. This is large enough to test the feasibility of the research procedures and to establish a mean and standard deviation on each outcome measure [42]. Five young people are to be randomised into the study each month. Recruitment rates and the final target will be used to inform the decision to proceed to a definitive trial.

Attrition will be minimised by having robust trial procedures to prevent data loss. Procedures have been developed with our Patient and Public Involvement (PPI) groups and tested for maintaining contact with participants. Recognising that this population can be difficult to follow-up, follow-up data collection points will occur within broad time windows. Having very broad time windows allows for as many follow-ups to be completed within the CYPSE, but also ensuring that no young people are excluded from the study if they miss a follow-up.

For peer review only - http://bmjopen.bmj.com/site/about/guidelines.xhtml

Randomisation and Blinding

Randomisation will be achieved by means of concealed random allocation conducted using an online pseudo-random list hosted by Sealedenvelope.com with random permuted blocks of varying sizes. Randomisation will be undertaken by the researcher, who will inform healthcare staff of trial arm allocation.

Given the nature of the intervention, it is not possible to blind participants or those involved in delivering the intervention. It is also not possible to blind the field researcher due to the researcher and staff delivering the intervention working within the confines of a secure environment. It is also not possible to use a second researcher to collect follow-up data (who does not know trial arm allocation) as this is logistically very challenging and ultimately conflicts with the idea of building a strong researcher-participant relationship to enhance follow-up rates. Therefore, to minimise any potential bias, protocolising the collection of outcome measures which will be overseen by the onsite Principal Investigator and trial manager (CL). None of the outcome measures completed by the young person with the researcher present are particularly susceptible to bias as they are standardised, self-complete questionnaires. The researcher may need to support the young person in completing questionnaires due to literacy levels and protocolising collection ensures that support is consistent and unbiased. The study statistician (LAC) will be blind to arm allocation.

Insert Table 1 about here

Data Collection

See table 1 for a summary of the study schedule of data collection.

Baseline data collection (t1)

The researcher will deliver the baseline data collection interview using a narrative conversational format. Demographics and questions from other measures are incorporated

For peer review only - http://bmjopen.bmj.com/site/about/guidelines.xhtml

into a specially constructed flexible paper Case Report Form which avoids duplication of subject matter in order to reduce disengagement or irritability.

Follow-Up (t2-t3)

If the young person is to be released during the study, the researcher will meet with them usually within the week prior to release. During this meeting, the researcher will confirm the contact information provided at baseline and make any amendments to the information (e.g. change of phone number). Once discharged the researcher will contact the participant again in the community, to maintain engagement, confirm contact details and arrange the follow-up in the community.

The 3-month follow-up can take place between 61 and 151 days post randomisation, although the researcher will endeavour to complete data collection as close to the 3 month (90 day) point. The 6-month follow-up can take place between 152 and 244 days post randomisation, although the researcher will endeavour to complete data collection close to the 6 month point (182 days).

The follow-up may take place in prison or in the community, depending on if the young person has been discharged. In the community, the researcher will arrange to meet the young person at a convenient location. Where possible, interviews will be conducted in the premises of services that the young person is engaged with in order to minimise risk to the researcher. Where this is not possible, the researcher will arrange to conduct the interviews in a suitable location in the community and adhere to the Local Lone Working Policy.

Process Evaluation

QbTest in itself may not be defined as a complex intervention requiring a process evaluation to understand the mechanisms of impact e.g. how QbTest produces change, however, delivering an intervention within a criminal justice setting is complex. Understanding the effects of the context as barriers or facilitators is critical for interpreting the findings and

For peer review only

For peer review only - http://bmjopen.bmj.com/site/about/guidelines.xhtml

generalising beyond it to a full trial. The process evaluation will follow the MRC guidelines [43] and will be conducted in parallel to the trial and will assess:

- the acceptability of randomisation (interviews);
- the acceptability of outcome measures (completeness of each measure and interviews);
- the numbers of young people agreeing to sit the QbTest and the number who complete QbTest;
- feedback from young people receiving the QbTest and health professionals using a questionnaire;
- the acceptability of the QbTest via interviews with young people; and
- health professionals and other CYPSE staff to obtain perspectives on facilitators and barriers to using the QbTest.

Data collection

QbTest completion rates: A record will be taken of the numbers of participants who agree to sit and also complete the QbTest.

QbTest opinion questionnaire: All young people who receive QbTest will be asked to complete the QbTest opinion questionnaire at 3 months. The questionnaire contains 11 questions e.g. ‘the QbTest results were difficult to understand’ and the young person is asked to rate each item on a 5-point scale. At month 15, clinicians involved in the QbTest will be asked to complete the clinician version, which contains 13 questions rated on a 5-point scale and two free text response questions [29].

Interviews with young people: Randomisation: A sub-sample of young people in both trial arms will be interviewed using a semi-structured interview schedule to further explore acceptability of the randomisation process. Young person’s age and time of randomisation (beginning or end of study) will be used to purposively select the sample. Outcomes: A sub-sample of young

For peer review only - http://bmjopen.bmj.com/site/about/guidelines.xhtml

people in both trial arms will be interviewed using a semi-structured interview schedule to further explore acceptability of completion of the outcome measures. Young person's age and time of randomisation (beginning or end of study) will be used to purposively select the sample. QbTest: A sub-sample of young people who receive QbTest will be interviewed using a semi-structured interview schedule to further explore acceptability of the QbTest and its administration as well as study processes and procedures. Scores/responses in the QbTest opinion questionnaire, young person's age and whether or not the QbTest was completed will be used to purposively select the sample. Up to 20 young people will be interviewed.

Interviews with healthcare professionals and CYPSE staff: Healthcare professionals and CYPSE staff will be invited to participate in qualitative interviews to further explore acceptability and feasibility of administering and implementing the QbTest within usual assessment practices and procedures, including the barriers to its use and reasons for non-completion. Scores/responses in the QbTest Opinion Questionnaire will be used to purposively select healthcare professionals. Up to 10 professionals will be interviewed.

Data Analysis and Management

Data analysis will be mainly descriptive [44]. All measures will be summarised by group across follow-up time with mean and standard deviation for normally distributed data, median for skewed variables and frequency (percentage) for categorical data. All statistical analysis will be conducted using Stata v.15. No interim analysis is planned.

Interviews will be conducted and recorded by the researcher using an encrypted Dictaphone. All qualitative data will be analysed using thematic analysis [45] with the aid of NVivo. Data will be coded inductively into themes, creating a detailed coding scheme, allowing for the investigation of emergent patterns between individual codes and boarder emergent themes.

For peer review only - http://bmjopen.bmj.com/site/about/guidelines.xhtml

Data will be double entered and stored on an encrypted and password protected database at the University of Manchester. Double-entered data will be compared for discrepancies and discrepant data will be verified using the original paper data sheets (between LWC and CL).

Monitoring

Adverse Events

Serious adverse events (SAEs) will be recorded by the research team and reported to the chief investigator and Trial Steering Committee (TSC). Any SAEs deemed to have a causal relationship to trial participation will be reported to the sponsor within 24 hours of the chief investigator being informed.

Trial Management and Oversight

The TSC will oversee the conduct and safety of the trial and includes the role of the data monitoring. The Committee includes an independent chair, independent members, a patient and public representative and the chief investigator. Representatives from both the Sponsor and funding organisations will be invited to study-related elements of the TSC meetings as observers. The TSC will meet quarterly.

Audit

The Trial Coordinator or a nominated designee of the Sponsor shall carry out monitoring of trial data as an ongoing activity. Trial data and evidence of monitoring and audits will be made available for inspection by the Research Ethics Committee as required.

For peer review only - http://bmjopen.bmj.com/site/about/guidelines.xhtml

ETHICS and DISSEMINATION

Health Research Authority approvals have been granted. Only the research team will have access to the study data, which will be stored in secure locked files or password-protected databases. Data will be available for inspection by the ethics committee on request. Changes to the protocol will be communicated to the ethics committee and trial registries by the trial manager (CL).

Consent Process

Obtaining informed consent or assent will be in accordance with ethical guidance and Good Clinical Practice. The researcher will be trained to assess for capacity to consent in young people. This will include assessing if the young person has the ability to understand the information relevant to the decision, retain the information, use or weigh the information as part of the process for decision making and communicate the decision to the researcher.

The researcher will explain what participation in the study involves and how much time will be involved. They will also explain that participation is voluntary, that they can withdraw at any time and at any point and that their decision to participate, or not, will have no adverse effect on the care that they receive or their other legal rights. The researcher will also discuss the arrangements in place to ensure confidentiality (and limits of this) and data protection. Throughout this process, the potential participant will be given an opportunity to ask questions. Potential participants will be made aware of circumstances in which confidentiality would be broken (if they or someone else identified may be at potential risk of harm; for example, self-harm, breaches of security and violence, including acts of terrorism/radicalisation).

For peer review only - http://bmjopen.bmj.com/site/about/guidelines.xhtml

Having had the opportunity to discuss their involvement in the study and ask questions about it, potential participants will be asked if they would like time (minimum 24 hours) to consider taking part or if they would like to sign the Consent Form (Supplementary file 1 – Consent Form) if they are willing to take part.

Patient and Public Involvement (PPI)

PPI significantly informs and impacts on this project. NB (co-applicant and co-author) is the project PPI representative and is directly involved in the project providing her lived experience expertise throughout the project ensuring that the perspective of young people with ADHD and their parents are considered throughout. The project has PPI representatives from the community and also within the CYPSE and both groups have helped develop the information leaflets for the study and also helped develop and refine research procedures e.g. how best to explain what participation in the study involves and how best to achieve community follow-ups. The CYPSE PPI group meet with the researcher (LWC) on a regular basis to feed into the project. The PPI groups will be involved in the analysis, reporting and dissemination of the research.

Dissemination

The findings from the trial will be used to inform the design, feasibility and acceptability of a future definitive trial. The findings will be published in peer-reviewed journals, presented at relevant conferences and disseminated to the public via summaries co-created with our PPI group.

For peer review only

For peer review only - http://bmjopen.bmj.com/site/about/guidelines.xhtml

DISCUSSION

Secure settings are complex environments in which to conduct trials [46] and there is a dearth of trials within the CYPSE. In the context of this challenging environment, a decision will be taken as to whether feasibility and acceptability are adequate for a full multi-centre randomised controlled trial.

Current service provision for ADHD in the CYPSE is ad hoc and uncoordinated. There is growing evidence within community settings [30, 32, 33, 34] that QbTest has the potential to significantly improve the current assessment pathway having a direct impact on young people's health, service delivery and criminal justice outcome.

For peer review only - http://bmjopen.bmj.com/site/about/guidelines.xhtml

Declarations

Ethics approval and consent to participate

This protocol (version 2) was approved by NHS Wales REC 3 (18/WA/0347) 15th February 2019 and HMPPS NRC 2018-315.

Consent for publication

Not applicable

Availability of data and material

Due to the population of research participants involved in this study individual-level data are not publicly available.

Competing interests

The authors declare that they have no competing interests.

Funding

The study is funded by the NIHR RfPB (Grant number: PB-PG-1216-20007).

For peer review only

Disclaimer

This paper presents independent research funded by the National Institute for Health Research (NIHR) under its Research for Patient Benefit (RfPB) Programme (Grant Reference Number PB-PG-1216-20007). The views expressed are those of the author(s) and not necessarily those of the NIHR or the Department of Health and Social Care.

Author Contributions

PC is the chief investigator and has overall responsibility for the design, conduct and decision to submit for publication. The study design was conceived by PC, CL (trial manager) and CH (co-investigator). CL and PC wrote the protocol with approval from all authors. LAC (co-investigator) is the study statistician and designed and wrote the analysis plan. LAC, CH, BB, SY, NB and AK are co-investigators on the NIHR RfPB grant and contributed to the study design, reviewed and commentated on the drafts of the manuscript. LWC & MR are the researchers collecting the trial data. All authors read and approved the final manuscript.

Trial Sponsor

Pennine Care NHS Foundation Trust; Reagan Blyth, Director of Service Modelling, Research and Innovation, Pennine Care NHS Foundation Trust, Trust Headquarters, 225 Old Street, Ashton-under-Lyne, OL6 7SR, reagan.blyth@nhs.net

For peer review only - http://bmjopen.bmj.com/site/about/guidelines.xhtml

References

1 Young S, Moss D, Sedgwick O, Fridman M, Hodgkins P. A meta-analysis of the prevalence of attention deficit hyperactivity disorder in incarcerated populations. Psychol Med. 2015; doi: 10.1017/S0033291714000762.

2 Baggio S, Fructuoso A, Guimaraes M, Fois E, Golay D, Heller P, et al. (2018) Prevalence of attention deficit hyperactivity disorder in detention settings: a systematic review and meta-analysis. Front Psychiatry. 2018; doi: 10.3389/fpsyt.2018.00331.

3 Hughes N, Williams H, Chitsabeasan P, Davies R, Mounce L. Nobody made the connection: The prevalence of neurodisability in young people who offend. The Children's Commissioner. 2012. https://www.childrenscommissioner.gov.uk/sites/default/files/publications/Nobody%20made%20the%20connection.pdf Accessed 17 May 2019.

4 Barkley RA. Attention-Deficit Hyperactivity Disorder: A Handbook for Diagnosis and Treatment. 3rd eds. New York: Guildford Press; 2006

5 Shaw M, Hodgkins P, Caci H, Young S, Kahle J, Wood AG et al. A systematic review and analysis of long-term outcomes in attention deficit hyperactivity disorder: effects of treatment and non-treatment. BMC Medicine. 2012; doi: 10.1186/1741-7015-10-99.

6 National Institute for Health and Care Excellence. Attention deficit hyperactivity disorder: diagnosis and management (CG72). National Institute for Health and Care Excellence.

For peer review only - http://bmjopen.bmj.com/site/about/guidelines.xhtml

2008. https://www.nice.org.uk/guidance/cg72?unlid=520684933201611441814 Accessed 17 May 2019.

7 National Institute for Health and Care Excellence. Attention deficit hyperactivity disorder (QS39). National Institute for Health and Care Excellence. 2013. https://www.nice.org.uk/guidance/qs39 Accessed 17 May 2019.

8 Takeda T, Ambrosini P, deBerardinis R, Elia J. What can ADHD without comorbidity teach us about comorbidity? Res Dev Disabil. 2012; doi: 10.1016/j.ridd.2011.09.024.

9 Moffitt TE. Juvenile delinquency and Attention Deficit Disorder: Developmental trajectories from age 3 to 15. Child Dev. 1990; 61: 893-910.

10 Moffitt TE. (1993). Adolescence-limited and life-course-persistent antisocial behavior: A developmental taxonomy. Psychol Rev. 1993; 100: 674-701.

11 Eme R. Attention deficit hyperactivity disorder and the family court. Fam Court Rev. 2009; doi: 10.1111/j.1744-1617.2009.01279.x.

12 Mental Health Taskforce. The five year forward view of mental health: A report from the independent Mental Health Taskforce to the NHS in England. 2016 https://www.england.nhs.uk/wpcontent/uploads/2016/02/Mental-Health-Taskforce-FYFV-final.pdf Accessed 17 May 2019.

13 Young S, Sedgwick O, Fridman M, Gudjonsson G, Hodgkins P, Lantigua M, et al. Comorbid psychiatric disorders amongst incarcerated ADHD populations: A meta-analysis. Psychol Med. 2015 doi: 10.1017/S0033291715000598.

14 Biederman J, Petty C, Clarke A, Lomedico A, Faraone SV. Predictors of persistent ADHD: An 11-year follow-up study. J Psychiatr Res. 2011; doi: 10.1016/j.jpsychires.2010.06.009.

15 Arnold LE, Hodgkins P, Kahle J, Madhoo M, Kewley G. Long-term outcomes of ADHD: Academic achievement and performance. J Atten Disord. 2015; pii: 1087054714566076.

16 D'Amico F, Knapp M, Beecham J, Sandberg S, Taylor E, et al. Use of services and associated costs for young adults with childhood hyperactivity/conduct problems: 20-year follow-up. Br J Psychiatry. 2014; doi: 10.1192/bjp.bp.113.131367.

For peer review only

For peer review only - http://bmjopen.bmj.com/site/about/guidelines.xhtml

17 Khong B.The lifetime costs of attention deficit hyperactivity disorder (ADHD). Centre for Mental Health. 2014. https://www.centreformentalhealth.org.uk/costs-of-adhd Accessed 17 May 2019.

18 Young S, Gonzalez R, Fridman M, Hodgkins P, Kim K, Gudjonsson G. The economic consequences of ADHD in the Scottish prison system. BMC Psychiatry. 2018. doi: 10.1186/s12888-018-1792-x.

19 Young S, González R, Gudjonsson GH. Health-related quality of life in prisoners with attention deficit/hyperactivity disorder and head injury. BMC Psychiatry. 2018; doi: 10.1186/s12888-018-1785-9.

20 Royal College of Paediatrics and Child Health. Healthcare standards for children and young people in secure settings. Royal College of Paediatrics and Child Health. 2013 https://www.rcpch.ac.uk/sites/default/files/2018-03/healthcare_standards_for_children_and_young_people_in_secure_settings_june_2013.pdf Accessed 17 May 2019.

21 Chitsabesan P, Lennox C, Theodosiou L, Law H, Bailey S, Shaw J. The development of the comprehensive health assessment tool for young offenders within the secure estate. J Forens Psychiatry Psychol. 2014; doi: 10.1080/14789949.2014.882387

22 Ministry of Justice. Youth justice annual statistics: 2014 to 2015. Ministry of Justice. 2016 https://www.gov.uk/government/statistics/youth-justice-annual-statistics-2014-to-2015 Accessed 17 May 2019.

23 Ministry of Justice. What works in managing young people who offend? Summary of the international evidence. Ministry of Justice. 2016. https://www.gov.uk/government/publications/whatworksin-managing-young-people-who-offend Accessed 17 May 2019.

24 Young S, Gudjonsson GH, Wells J, Asherson P, Theobald D, et al. Attention deficit hyperactivity disorder and critical incidents in a Scottish prison population. Pers Individ Dif. 2009; doi: 10.1016/j.paid.2008.10.003.

For peer review only

For peer review only - http://bmjopen.bmj.com/site/about/guidelines.xhtml

25 Young S, Misch P, Collins P, Gudjonsson GH. Predictors of institutional behavioural disturbance and offending in the community among young offenders. J Forens Psychiatry Psychol. 2011; doi: 10.1080/14789949.2010.495991.

26 Ministry of Justice. Safety in Custody Statistics Bulletin: Deaths in prison custody to September 2016; Assaults and self-harm to June 2016. Ministry of Justice. 2016. https://www.gov.uk/government/uploads/system/uploads/attachment_data/file/562897/safety-incustodybulletin.pdf Accessed 17 May 2019.

27 Lichtenstein P, Halldner L, Zetterqvist J, Sjolander A, Serlachius E, Fazel S, et al. Medication for attention deficit-hyperactivity disorder and criminality. N Engl J Med. 2012; doi: 10.1056/NEJMoa1203241.

28 Reh V, Schmidt M, Lam L, Schimmelmann BG, Hebebrand J, Rief W, et al. Behavioral assessment of core ADHD symptoms using the QbTest. J Atten Disord. 2015; doi: 10.1177/1087054712472981.

29 Reh V, Schidt M, Rief W, Christiansen H. Preliminary evidence for altered motion tracking-based hyperactivity in ADHD siblings. Behavl Brain Funct. 2014; doi: 10.1186/1744-9081-10-7.

30 Hall CL, Valentine AZ, Groom MJ, Walker GM, Daley D, Hollis C. The clinical utility of the continuous performance test and objective measures of activity for diagnosing and monitoring ADHD in children: a systematic review. Eur Child Adolesc Psychiatry. 2016. doi: 10.1007/s00787-015-0798-x.

31 Vogt C, Shameli A. Assessments for attention-deficit hyperactivity disorder: use of objective measurements. Psychiatrist. 2011; doi: 10.1192/pb.bp.110.032144

32 Hall C, Selby K, Boliang G, Althea V, Walker G, Hollis C. Innovations in Practice: an objective measure of attention, impulsivity and activity reduces time to confirm attention deficit/hyperactivity disorder diagnosis in children – a completed audit cycle. Child Adolesc Ment Health. 2016; doi: 10.1111/camh.12140

33 Hollis C, Hall CL, Guo B, James M, Boadu J, Groom M, et al. The impact of a computerised test of attention and activity (QbTest) on diagnostic decision-making in children and young people with suspected attention deficit hyperactivity disorder: single-

For peer review only

blind randomised controlled trial. J Child Psychol Psychiatry. 2018; doi:10.1111/jcpp.1292.1

34 Hall C, Valentine AZ, Walker GM, Ball HM, Cogger H, Daley D, et al. Study of user experience of an objectice test (QbTest) to aid ADHD assessement and medication management: a multi-methods approach. BMC Psychiatry. 2017; doi: 10.1186/s12888-017-1222-5.

35 Medical Research Council. Developing and evaluating complex interventions: new guidance. Medical Research Council. 2008. http://www.mrc.ac.uk/documents/pdf/complexinterventionsguidance/ Accessed 17 May 2019.

36 Goodman R. The Strengths and Difficulties Questionnaire: A research note. J Child Psychol Psychiatry. 1997; 38: 581–586

37 Young S, González RA, Mutch L, Mallet-Lambert I, O'Rourke L. et al. Diagnostic accuracy of a brief screening tool for attention deficit hyperactivity disorder in UK prison inmates. Psychol Med. 2016; doi: 10.1017/S0033291716000039.

38 Stevens KJ. Assessing the performance of a new generic measure of health related quality of life for children and refining it for use in health state valuation. Appl Health Econ Health Policy. 2011; doi: 10.2165/11587350-000000000-00000.

39 Swanson JM, Kraemer HC, Hinshaw S P, Arnold LE, Conners CK, Abikokk HB, et al. Clinical relevance of the primary findings of the MTA: success rates based on severity of ADHD and ODD symptoms at the end of treatment. J Am Acad Child Adolesc Psychiatry. 2001; 40: 168-79.

40 Shaffer N, Gould MS, Brasic J. A children's Global Assessment Scale (CGAS). Arch Gen Psychiatry. 1983; 40:1228-1231.

41 Beecham J, Knapp M. Costing psychiatric interventions. In G. Thornicroft (ed.) Measuring Mental Health Needs, Gaskell, 2nd edition, p200-224. 2001.

42 Hertzog MA. Considerations in determining sample size for pilot studies. Res Nurs Health. 2008; doi: 10.1002/nur.20247.

For peer review only

43 Moore G, Audrey S, Barker M, Bond L, Bonell C. Hardman W, et al. Process evaluation of complex interventions: Medical Research Council guidance. BMJ. 2015; doi: 10.1136/bmj.h1258.

44 Lancaster GA, Dodd SR, Williamson PR: Design and analysis of pilot studies: recommendations for good practice. J Eval Clin Pract. 2004, doi: 10.1111/j..2002.384.doc.x.

45 Braun V, Clarke V. Using thematic analysis in psychology. Qual Res Psychol. 2006; 3: 77-101.

46 Pettus-Davis C, Howard MO, Dunningan A, Scheyett AM, Roberts-Lewis A. Using randomized controlled trial to evaluate interventions for releasing prisoners. Res Soc Work Pract. 2015; doi: 10.1177/1049731515579203

Table 1: Summary of study schedule

		Screening	Baseline	Allocation	Follow-Up	
**TIMEPOINT**		***t0***	***t1***		***+3 mth t2***	***+6 mth t3***
**ENROLMENT:**						
**Eligibility screen**		X				
**Informed consent**		X				
Parental consent (< 16)		X				
**Allocation**				X		
**INTERVENTIONS:**						
Intervention Group:	***QbTest***					
	Usual care					
Control Group:	***Usual care***					
**ASSESSMENTS:**						
Demographics			X		X	X
SDQ			X		X	X
B-BAARS			X		X	X
CHU-9D			X		X	X
SNAP-IV			X		X	X
C-GAS			X		X	X
ADHD Diagnosis & Clinical Confidence					X	X
Health Records			X		X	X
Prison Records			X		X	X

For peer review only - http://bmjopen.bmj.com/site/about/guidelines.xhtml

Modified CSRI		X		X	X
Contact Sheet		X		X	
**SAFETY MONITORING:**					
Adverse event reporting	◆————————————◆				

SDQ = Strengths and Difficulties Questionnaire; B-BAARS = Brief Barkley ADHD Rating Scale; CHU-9D = Child Health Utility Instrument; SNAP-IV = Swanson, Nolan and Pelham Questionnaire; C-GAS = Children's Global Assessment Scale; CSRI = Client Service Receipt Inventory

For peer review only

Pennine Care NHS Foundation Trust

Leeds Community Healthcare NHS Trust

FACT: Feasibility trial to assess ADHD in the Criminal Justice System via QBTest

PARTICIPANT CONSENT FORM

IRAS project ID: 238947

Please **initial**

1. I confirm that I have read the attached information sheet (**Version X, Date: dd/mm/yy**). ☐

2 I have had time to think about the information, ask questions and had these answered. ☐

3. I understand that taking part in the study is up to me and that I am free to stop taking part at any time without giving a reason. This will not affect my care or cause problems for me. ☐

4. I understand that the researcher has to tell prison staff should I talk about: ☐
   a) Serious behaviour that is against prison rules;
   b) Harm to myself or others;
   c) Plans to commit new crimes or past crimes that the police do not know about;
   d) Information that raises concerns about terrorist, radicalisations, or security issues.

5. I understand that I will be in the QbTest group or usual care group. My group will be chosen at random, and I won't be able to choose. ☐

6. I agree to the research team looking at my criminal justice and health records, but only for the purpose of this research project. ☐

7. I agree to interviews being audio recorded. ☐

8. I agree that direct quotes can be used in any study write-up, in anonymous form. ☐

9. I agree that any data collected may be published in anonymous form in academic books, reports or journals ☐

10. I agree that the researcher team can have my contact details so that they can contact me for the 3 and 6 month follow up interviews. ☐

11. I agree to take part in this study ☐

Sign below for participant completed consent form

Name of participant	Date	Signature
Name of researcher	Date	Signature

(Version 2, Date: 25.10.18)

For peer review only - http://bmjopen.bmj.com/site/about/guidelines.xhtml

Pennine Care NHS Foundation Trust

Leeds Community Healthcare NHS Trust

*1 copy for participant: 1 copy for researcher: 1 copy for the participant record

Data Protection - The personal information we collect and use to conduct this research will be processed in accordance with data protection law as explained in the Participant Information Sheet.

For peer review only

For peer review only - http://bmjopen.bmj.com/site/about/guidelines.xhtml

STANDARD PROTOCOL ITEMS: RECOMMENDATIONS FOR INTERVENTIONAL TRIALS

SPIRIT 2013 Checklist: Recommended items to address in a clinical trial protocol and related documents*

Section/item	Item No	Description	Addressed on page number
**Administrative information**			
Title	1	Descriptive title identifying the study design, population, interventions, and, if applicable, trial acronym	P 1; L 1&2
Trial registration	2a	Trial identifier and registry name. If not yet registered, name of intended registry	P 3; L77
	2b	All items from the World Health Organization Trial Registration Data Set	n/a
Protocol version	3	Date and version identifier	P 21; L581
Funding	4	Sources and types of financial, material, and other support	P 21; L595
Roles and responsibilities	5a	Names, affiliations, and roles of protocol contributors	P 21 & 22; L 604-611
	5b	Name and contact information for the trial sponsor	P 22; L 614-616
	5c	Role of study sponsor and funders, if any, in study design; collection, management, analysis, and interpretation of data; writing of the report; and the decision to submit the report for publication, including whether they will have ultimate authority over any of these activities	P 21; L597-600
	5d	Composition, roles, and responsibilities of the coordinating centre, steering committee, endpoint adjudication committee, data management team, and other individuals or groups overseeing the trial, if applicable (see Item 21a for data monitoring committee)	P17; L468-472

For peer review only

**Introduction**			
Background and rationale	6a	Description of research question and justification for undertaking the trial, including summary of relevant studies (published and unpublished) examining benefits and harms for each intervention	P5-7; L118-201
	6b	Explanation for choice of comparators	n/a
Objectives	7	Specific objectives or hypotheses	P 8; L205&206
Trial design	8	Description of trial design including type of trial (eg, parallel group, crossover, factorial, single group), allocation ratio, and framework (eg, superiority, equivalence, noninferiority, exploratory)	P8; 209-212
**Methods: Participants, interventions, and outcomes**			
Study setting	9	Description of study settings (eg, community clinic, academic hospital) and list of countries where data will be collected. Reference to where list of study sites can be obtained	P 9; L236
Eligibility criteria	10	Inclusion and exclusion criteria for participants. If applicable, eligibility criteria for study centres and individuals who will perform the interventions (eg, surgeons, psychotherapists)	P 9; L 239-262
Interventions	11a	Interventions for each group with sufficient detail to allow replication, including how and when they will be administered	P10; L 264- 283
	11b	Criteria for discontinuing or modifying allocated interventions for a given trial participant (eg, drug dose change in response to harms, participant request, or improving/worsening disease)	n/a
	11c	Strategies to improve adherence to intervention protocols, and any procedures for monitoring adherence (eg, drug tablet return, laboratory tests)	n/a
	11d	Relevant concomitant care and interventions that are permitted or prohibited during the trial	n/a
Outcomes	12	Primary, secondary, and other outcomes, including the specific measurement variable (eg, systolic blood pressure), analysis metric (eg, change from baseline, final value, time to event), method of aggregation (eg, median, proportion), and time point for each outcome. Explanation of the clinical relevance of chosen efficacy and harm outcomes is strongly recommended	P10-12; L285- 326
Participant timeline	13	Time schedule of enrolment, interventions (including any run-ins and washouts), assessments, and visits for participants. A schematic diagram is highly recommended (see Figure)	Table 1; P28; L778

For peer review only - http://bmjopen.bmj.com/site/about/guidelines.xhtml

Sample size	14	Estimated number of participants needed to achieve study objectives and how it was determined, including clinical and statistical assumptions supporting any sample size calculations	P12; L329-333
Recruitment	15	Strategies for achieving adequate participant enrolment to reach target sample size	P12; L335-340
**Methods: Assignment of interventions (for controlled trials)**			
Allocation:			
Sequence generation	16a	Method of generating the allocation sequence (eg, computer-generated random numbers), and list of any factors for stratification. To reduce predictability of a random sequence, details of any planned restriction (eg, blocking) should be provided in a separate document that is unavailable to those who enrol participants or assign interventions	P12; L343-346
Allocation concealment mechanism	16b	Mechanism of implementing the allocation sequence (eg, central telephone; sequentially numbered, opaque, sealed envelopes), describing any steps to conceal the sequence until interventions are assigned	P12; L343-346
Implementation	16c	Who will generate the allocation sequence, who will enrol participants, and who will assign participants to interventions	P12; L343-346
Blinding (masking)	17a	Who will be blinded after assignment to interventions (eg, trial participants, care providers, outcome assessors, data analysts), and how	P 13; L348-361
	17b	If blinded, circumstances under which unblinding is permissible, and procedure for revealing a participant's allocated intervention during the trial	n/a
**Methods: Data collection, management, and analysis**			
Data collection methods	18a	Plans for assessment and collection of outcome, baseline, and other trial data, including any related processes to promote data quality (eg, duplicate measurements, training of assessors) and a description of study instruments (eg, questionnaires, laboratory tests) along with their reliability and validity, if known. Reference to where data collection forms can be found, if not in the protocol	P13&14; L364-392
	18b	Plans to promote participant retention and complete follow-up, including list of any outcome data to be collected for participants who discontinue or deviate from intervention protocols	P13&14; L364-392

Data management	19	Plans for data entry, coding, security, and storage, including any related processes to promote data quality (eg, double data entry; range checks for data values). Reference to where details of data management procedures can be found, if not in the protocol	P16; L455-458
Statistical methods	20a	Statistical methods for analysing primary and secondary outcomes. Reference to where other details of the statistical analysis plan can be found, if not in the protocol	P16; L445-448
	20b	Methods for any additional analyses (eg, subgroup and adjusted analyses)	n/a
	20c	Definition of analysis population relating to protocol non-adherence (eg, as randomised analysis), and any statistical methods to handle missing data (eg, multiple imputation)	n/a
**Methods: Monitoring**			
Data monitoring	21a	Composition of data monitoring committee (DMC); summary of its role and reporting structure; statement of whether it is independent from the sponsor and competing interests; and reference to where further details about its charter can be found, if not in the protocol. Alternatively, an explanation of why a DMC is not needed	P17; L468-472
	21b	Description of any interim analyses and stopping guidelines, including who will have access to these interim results and make the final decision to terminate the trial	n/a
Harms	22	Plans for collecting, assessing, reporting, and managing solicited and spontaneously reported adverse events and other unintended effects of trial interventions or trial conduct	P16-17; L461-465
Auditing	23	Frequency and procedures for auditing trial conduct, if any, and whether the process will be independent from investigators and the sponsor	P17; L472
**Ethics and dissemination**			
Research ethics approval	24	Plans for seeking research ethics committee/institutional review board (REC/IRB) approval	P18, L491-496; P21; L581-582
Protocol amendments	25	Plans for communicating important protocol modifications (eg, changes to eligibility criteria, outcomes, analyses) to relevant parties (eg, investigators, REC/IRBs, trial participants, trial registries, journals, regulators)	P18; L495-496

For peer review only - http://bmjopen.bmj.com/site/about/guidelines.xhtml

Consent or assent	26a	Who will obtain informed consent or assent from potential trial participants or authorised surrogates, and how (see Item 32)	P18; L498-518
	26b	Additional consent provisions for collection and use of participant data and biological specimens in ancillary studies, if applicable	n/a
Confidentiality	27	How personal information about potential and enrolled participants will be collected, shared, and maintained in order to protect confidentiality before, during, and after the trial	P18; L508-514
Declaration of interests	28	Financial and other competing interests for principal investigators for the overall trial and each study site	P21; L592
Access to data	29	Statement of who will have access to the final trial dataset, and disclosure of contractual agreements that limit such access for investigators	P21; L588-589
Ancillary and post-trial care	30	Provisions, if any, for ancillary and post-trial care, and for compensation to those who suffer harm from trial participation	n/a
Dissemination policy	31a	Plans for investigators and sponsor to communicate trial results to participants, healthcare professionals, the public, and other relevant groups (eg, via publication, reporting in results databases, or other data sharing arrangements), including any publication restrictions	P19; L533-536
	31b	Authorship eligibility guidelines and any intended use of professional writers	n/a
	31c	Plans, if any, for granting public access to the full protocol, participant-level dataset, and statistical code	P21; L588-589
**Appendices**			
Informed consent materials	32	Model consent form and other related documentation given to participants and authorised surrogates	n/a
Biological specimens	33	Plans for collection, laboratory evaluation, and storage of biological specimens for genetic or molecular analysis in the current trial and for future use in ancillary studies, if applicable	n/a

*It is strongly recommended that this checklist be read in conjunction with the SPIRIT 2013 Explanation & Elaboration for important clarification on the items. Amendments to the protocol should be tracked and dated. The SPIRIT checklist is copyrighted by the SPIRIT Group under the Creative Commons "Attribution-NonCommercial-NoDerivs 3.0 Unported" license.

For peer review only - http://bmjopen.bmj.com/site/about/guidelines.xhtml
