# Supplementary material for: FACT: a randomised controlled trial to assess the feasibility of QbTest in the assessment process of attention deficit hyperactivity disorder (ADHD) for young people in prison—a feasibility trial protocol
Source: BMJ Open. 2020 Jan 20;10(1):e035519. doi: 10.1136/bmjopen-2019-035519 (PMC7044874; doi:10.1136/bmjopen-2019-035519)
Supplement: Reviewer comments [file bmjopen-2019-035519.reviewer_comments.pdf]

PEER REVIEW HISTORY

BMJ Open publishes all reviews undertaken for accepted manuscripts. Reviewers are asked to complete a checklist review form (**http://bmjopen.bmj.com/site/about/resources/checklist.pdf**) and are provided with free text boxes to elaborate on their assessment. These free text comments are reproduced below.

ARTICLE DETAILS

**TITLE (PROVISIONAL)**	FACT: A randomised controlled trial to assess the feasibility of QbTest in the assessment process of attention deficit hyperactivity disorder (ADHD) for young people in prison: A feasibility trial protocol.
**AUTHORS**	Lennox, Charlotte; Hall, Charlotte Lucy; Carter, Lesley-Anne; Beresford, Bryony; Young, Susan; Kraam, Abdullah; Brown, Nikki; Wilkinson-Cunningham, Lloyd; Reeves, Mindy; Chitsabesan, Prathiba

This article was not externally reviewed at BMJ Open. Protocols that have been independently assessed prior to submission to BMJ Open are usually fast-tracked to publication on the grounds that further substantial changes will not be possible. This independent assessment will usually be external, independent review for both a substantial grant award from a non-commercial or government funder and ethics approval.
